# Structural characterization and *in-silico* analysis of *Momordica charantia* 7S globulin for stability and ACE inhibition

**DOI:** 10.1038/s41598-020-58138-9

**Published:** 2020-01-24

**Authors:** Pooja Kesari, Shivendra Pratap, Poonam Dhankhar, Vikram Dalal, Manisha Mishra, Pradyumna Kumar Singh, Harsh Chauhan, Pravindra Kumar

**Affiliations:** 1 Department of Biotechnology, Indian Institute of Technology Roorkee, Roorkee, Uttarakhand 247667 India; 2 Plant Molecular Biology Division, Council of Scientific and Industrial Research (CSIR)-National Botanical Research Institute, Lucknow, India

**Keywords:** Protein function predictions, X-ray crystallography

## Abstract

*Momordica charantia* (*Mc*) seeds are widely used edible crop with high nutritional quality. The food and pharmaceutical industries use it as a natural anti-oxygenic agent. Herein, a ~52 kDa protein, which is a major part of seed proteome has been purified, biochemically characterized and structure has been determined. MALDI-ESI-MS identified peptide fragments and contig-deduced sequence suggested the protein to be homologous to 7S globulins. The crystal structure shows that protein has a bicupin fold similar to 7S globulins and the electron density for a copper and acetate ligand were observed in the C-terminal barrel domain. *In silico* study reveals that a tripeptide (VFK) from *Mc*7S possess a higher binding affinity for angiotensin converting enzyme (ACE) than already reported drug Lisinopril (LPR). The protein is a glycoprotein and highly stable under varying thermal and pH conditions due to its secondary structures. The DPPH (2,2-diphenyl-1-picryl-hydrazyl-hydrate) assay showed the protein to have an anti-oxygenic nature and can aid in scavenging free radical from sample. The protein can assist to enhance the nutritional and functional value of food by acting as a food antioxidant. Further, characterization of *Mc*7S required which might add in importance of *Mc*7S as antioxidant, anti-diabetic and anti-hypertensive.

## Introduction

*Momordica charantia (Mc)* commonly known as bitter melon or bitter gourd is a member of Cucurbitaceae family, which grows in tropical and sub-tropical areas. The plant is known for centuries in Ayurveda for its anti-bacterial, anti-fungal, anti-viral, anti-parasitic, hypoglycemic, anti-fertility, anti-tumorous, and anti-carcinogenic properties^1–5^. It is preferred as a medication for a broad range of health applications, including the treatment of dysmenorrhea, eczema, emmenagogue, galactagogue, gout, jaundice, kidney (stone), leprosy, leucorrhea, piles, pneumonia, psoriasis, rheumatism, scabies, T2DM (type 2 diabetes mellitus), obesity, hypertension, bacterial and viral infections, cancer, and even AIDS^1–5^. It contains biologically active molecules including proteins, triterpenes, saponins, steroids, flavonoids, alkaloids, and acids^6^. Each part of plant i.e. seeds, roots, leaves and especially unripe fruits has its pharmacological properties^4^. The juice is used to cure a large number of conditions such as for articular pain relief and chronic fever in jaundice, liver disease, and digestive system diseases because of laxative, diuretic, and anti-helminthic effects. In the case of chronic skin diseases, it is applied locally to treat boils, burns, and rash. Moreover, components of *Mc* plant i.e. unripe fruit, seeds and aerial parts are largely known for its anti-diabetic properties due to the presence of insulin-mimetic in the seeds^7,8^. The aqueous seed extract of *Mc* also scavenges the free radicals for protection against lipid peroxidation thereby reducing the risk of diabetic complications^9^. The strong anti-oxygenic activity may be due to the presence of phenolic compounds and saponins^10^.

The globular seed fraction of *Mc* majorly contains globulins, which are high-molecular weight proteins, initially thought to be catalytically inactive, however, with the passage of time; new activities have been associated with these proteins. Globulins belong to cupin superfamily and are categorized as 11–12S legumin and 7–8S vicilin based on its sedimentation coefficients. Vicilins are trimeric protein expressed by multiple structural genes and their extensive post-translational processing (glycosylation and protease action) results in a high degree of polymorphism^11^. The expression of 7S globulins increases under dehydrating conditions and therefore they are thought to be involved in desiccation and oxidative stress as well^12^.

In this study, we have structurally and functionally characterized a 52 kDa protein from the seeds of *Mc*. The MALDI-ESI-MS characterized fragments suggest the protein has homology with 7S globulin. In the absence of 7S globulin sequence, the contig sequences were aligned to deduce the protein sequence. The crystal structure of *Momordica charantia* 7S (*Mc*7S) globulin showed maximum homology to 7S vicilins. The structural analysis led to the identification of a bound copper and acetate ion in the protein. *In silico* study indicates that *Mc*7S release the peptide (VFK) which can bind with angiotensin-converting enzyme (ACE). Further, molecular docking and simulation confirm that binding of VFK to ACE tends to form stable ACE-VFK complex. Protein is highly stable upto 85 °C, so this protein can be used in food industry. Independently, the protein also possesses antioxidant activity and is glycosylated. The protein plays a significant role in food system and human health system by reducing oxidative stress and free radicals.

## Results

### Purification of *Mc*7S globulin

*Mc*7S globulin was purified to homogeneity by a three-step procedure involving ammonium sulphate precipitation, affinity chromatography and size exclusion chromatography. In ammonium sulphate precipitation step, 7S globulin was fractionated in 60–80% fraction, as observed on SDS-PAGE. This fraction showed two major protein bands of ~52 kDa and ~33 kDa and further, taken for affinity purification using Affi-Gel Blue matrix and the desired protein was eluted with a stepwise gradient of NaCl. The ~52 kDa protein was eluted in-between the 0.2 M–0.5 M NaCl fractions. The eluted fractions were pooled and dialyzed; before applying onto HiLoad 16/60 Superdex 200 pg column. The purity of ~52 kDa protein fraction was confirmed by the presence of a single band on SDS–PAGE gel and the Gel filtration chromatogram showed peak at ~69.2 ml (Fig. S1). The molecular weight of protein was estimated by using HMW calibration curve, which suggests that the protein exists as a trimer of ~156 kDa.

### Sequence analysis

To identify the ~52 kDa protein isolated from the seeds, the protein was analysed using MALDI-ESI-MS. Three peptide fragments FAILEAR, PHFNSR and LVGFGINAQNNLR were identified (Fig. S2). The derived peptide sequences were searched against the EST database and protein sequence database. The literature suggests that the protein could be a seed storage protein; however no hit was obtained against the protein sequence database, therefore the transcriptome data of *Mc* was analysed to identify cDNA population encoding 7S globulin. The previously reported literature on the sequencing of bitter gourd results suggested that 42 contigs of bitter gourd share high sequence homology with cupin 2, PV100 from *Cucurbita maxima* (gi|3808062). These contigs were aligned using DNAStar software package. Each aligned fragment was searched against the nucleotide database and protein database to identify the nearest homologs. The contig-deduced nucleotide sequence was translated to the protein sequence. BLAST result against protein database showed that the derived protein sequence which shares the maximum homology with cupin 2, PV100 (gi|3808062) of cupin superfamily and shares 73% homology with vicilin from *Cucumis sativus* (XP_011650968.1). The alignment of contigs resulted in the identification of 419 amino acid residues having a molecular weight of ~47 kDa and theoretical pI of 7.22. Supplementary Data SD1 shows the alignment of few contig fragments. The composition of 20 amino acids of *Mc*7S, vicilin from *Solanum* and *Capsicum* is given in Table S1. The protein has a large number of charged residues and very few cysteine and tryptophan residues. It is rich in essential amino acids like phenylalanine, valine, threonine, leucine, isoleucine and lysine.

### Crystal structure of *Mc*7S

Purified protein was concentrated to about 10 mg/ml and immediately used for crystallization. Diffraction-quality crystals of protein were obtained 0.1 M sodium acetate trihydrate pH 4.6 and 2 M sodium chloride. The crystal structure of protein was determined at a final resolution of 3.1 Å. It belongs to *P*63 space group and has a single monomer in an asymmetric unit. The chain A of the crystal structure of vicilin from pecan (*Carya illinoinensis*) (PDB ID: 5E1R) which share 52% sequence similarity with *Mc*7S was used as a template for model building. Since the template sequence has good query coverage the initial R_factor_ and R_free_ values were 0.31 and 0.41. The final R_factor_ and R_free_ values after iterative cycles of restrained refinement and model building were 0.19 and 0.27, respectively. The numbering of *Mc*7S residues is done as per canavalin. The Ramachandran plot generated using the MolProbity server for analysis of the main chain stereochemistry showed that 90.7% residues are in the favoured region and 9% residues in allowed region and 0.3% are in outlier of the plot. The final statistics of data collection and refinement parameters have been described in Table 1.

**Table 1:** Data collection and refinement statistics of *Mc*7S.

PDB ID	6KM8
Wavelength (Å)	1.5418
Resolution range (Å)	50–3.1 (3.15–3.1)
Space group	*P* 6_3_
**Cell dimensions**	
*a, b, c* (Å), *α, β, γ* (°)	90.77, 90.77, 68.48, 90, 90, 120
Total no of Reflections	27725
Unique reflections	5314 (397)
Multiplicity	2.8 (2.1)
Completeness (%)	89.52 (66.44)
Mean I/sigma(I)	11.17 (2.29)
R-sym	0.12 (0.407)
R-factor	0.19
R- free	0.27
RMSD bonds lengths (Å)	0.01
RMSD bond angles (°)	1.50
Number of non-hydrogen atoms	2753
Macromolecules	2741
Number of protein residues	345
Ramachandran favored (%)	90.7
Ramachandran Allowed (%)	9.0
Ramachandran outliers (%)	0.3
Wilson B factor (Å^2^)	53.8
Average B-factor (Å^2^)	53.0

Statistics for the highest-resolution shell are shown in parentheses.

R_merge_ = Σ_hkl_Σ_j_|*I*_hkl,j_ − < *I*_hkl_ > |/Σ_hkl_Σ_j_|*I*_hkl,j_.

#### Overall structure of *Mc*7S

**Figure 1:**
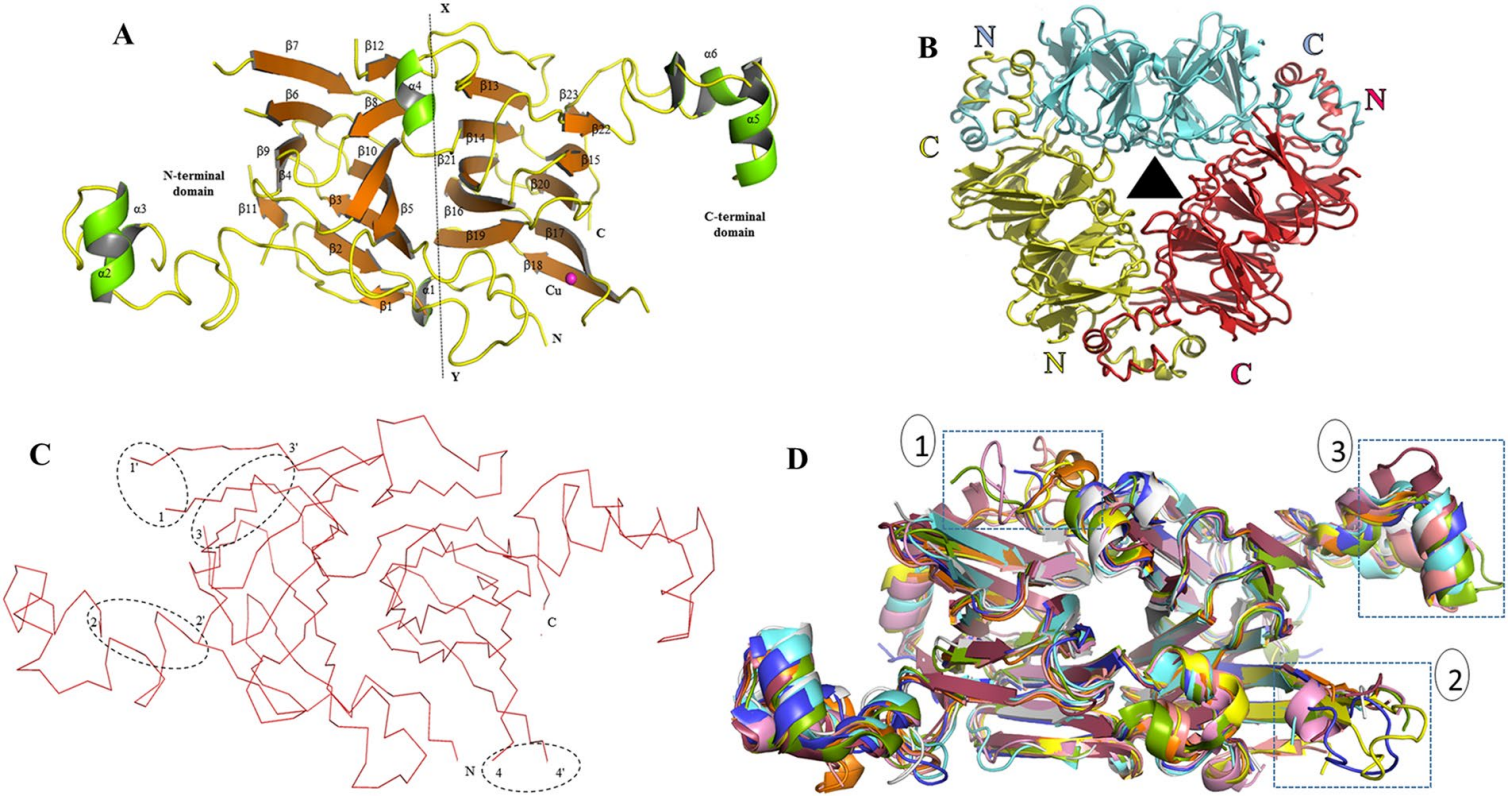
Crystal structure of *Mc*7S globulin. (**A**) The crystallographic asymmetric unit of *Mc*7S globulin contains a monomer which is represented by a cartoon form. In the monomer, the strands, helixes, and random coils are shown in orange, green, and yellow, respectively. The pseudo-dyad axis of symmetry (X-Y axis) divides the monomer into N and C-terminal domain. Each domain is comprised of a cupin fold. The N and C-terminal ends have been marked as N and C. The β sheets and α-helices have been labelled as per occurrence. The magenta sphere is the Cu ion. (**B**) The biological *Mc*7S trimer assembly is formed by interaction of monomers in head-to-tail fashion. The 3 chains of *Mc*7S is represented in cartoon form in green, pink and purple, respectively. The hydrophobic surface residues are coloured in red. The triangle in the centre represents the axis perpendicular to the three-fold axis of symmetry. (**C**) Four disordered region of *Mc*7S (I: 125–127 residues, II: 215–220 residues, III: 225–247 residues, and IV: 325–334 residues) have been circled. The ending residues of the disordered loop is marked by apostrophe (‘). (**D**) Structural superimposition of vicilin from *Solanum melongena* SM80.1 (5vf5-golden), pecan (cyan), AraH1 (magenta), Korean pine (green), β-conglycinin (orange), phaseolin (brown), 8S mungbean (purple), Aduzki bean (light red), Jackbean (blue), *Capsicum annum* Vic_CAPAN (pink) over *Mc*7S (white). The region of variation is marked by blue dotted box. Box 1 represent the region around disorder loop 3, the box 2 is the copper binding site whereas the box 3 is the C-terminal helical region.

The monomer of *Mc*7S comprises of two cupin fold polypeptide chain separated by a pseudo-dyad axis of symmetry as observed in the other 7S family members (Fig. 1A). This pseudo-dyad axis divides monomer into two domains, namely N- and C-terminal domains. The core region of the N- and C-terminal domains are composed of β-sheets whereas the helices form the extended arms. The N-terminal barrel fold (residues 46–248) is composed of 11 β sheets, i.e. β1 (Ser56-Ser58), β2 (Gly65-Leu70), β3 (Arg87-Ala93), β4 (Thr97-Ile99), β5 (Ala106-Val112), β6 (Ala116-Val123), β7 (Lys128-Ala136), β8 (Gly139-Ile144), β9 (Thr149-Val153), β10 (Gln161-Pro168) and β11 (Asp177-Leu179) surrounded by two extended α-helices α2 (Asn194-Leu201), α3 (Arg207-Val211). Similarly C-terminal barrel fold (residues 265–426) is composed of 12 β sheets β12 (Leu250-Lys252), β13 (Glu265-Ala270), β14 (Ala284-Ile290), β15 (Gly295-Asn301), β16 (Thr305-Glu312), β17 (Ser316-Cys321), β18 (Glu337-His342), β19 (Leu348-Val351), β20 (Leu367-Met360), β21 (Leu367-Ile374), β22 (Arg381-Phe383) and β23 (Phe422-Lys424) surrounded by two extended α-helices α5 (Gln396–Thr402), α6 (Lys408-Glu412). The sequence alignment of N- and C- terminal domain of *Mc*7S suggests that both domains share an evolutionary relationship (Fig. S3). There are around 44 and 49 residues on the interface between the N-terminal and C-terminal domains, respectively which form 18 hydrogen bonds and 8 salt bridges (Table S2).

As per gel filtration profile, the *Mc*7S globulin should exist as a trimer. The trimer was observed when the three monomer molecules were arranged in the form of an equilateral triangle where their extended α-helix regions interact with the other monomer in a head-to-tail fashion (Fig. 1B). For the formation of the trimer, the monomers are arranged in a three-fold axis of symmetry. Similar fold was also observed in reported 7S seed storage proteins, i.e. adzuki bean 7S globulin (PDB ID: 2EAA and 2EA7), jackbean canavalin (PDB ID: 2CAU), french bean phaseolin (PDB ID: 2PHL), 8S mungbean (PDB ID: 2CV6), Korean pine (PDB ID: 4LEJ), pecan (PDB ID: 5E1R), peanut AraH1 (PDB ID: 3SMH), soybean β-conglycinin (PDB ID: 1UIK), *Capsicum annum* Vic_CAPAN (PDB ID: 5YJS) and *Solanum melongena* SM80.1 (PDB ID: 5VF5). Thus, the trimer was generated by applying the cell symmetry in coot, showing three molecules from three asymmetric unit. The hydrophobicity of monomer of globulin is higher as compared to other seed storage protein^13^. However, these surface hydrophobic residues get buried inside during head-to-tail interaction of the tertiary structure. Also, charged residues from the extended helical arm of the monomer are interlocked in the tertiary structure through salt-bridges and hydrogen bonding. The interface of chain A-chain B forms 25 hydrogen bonds and 4 salt bridges whereas the interface of chain C-chain A form 25 hydrogen bonds and 4 salt bridges (Table S3).

In *Mc*7S, the density was observed for 342 residues of the monomer out of 383 residues. Four stretches (I: 125–127 residues, II: 215–220 residues, III: 225–247 residues, and IV: 325–334 residues) were omitted from the final model because no electron density at the main-chain level was available (Fig. 1C).

#### Sequence and structural comparison with other 7S seed storage globulin

The 7S globulins or vicilins share ~40% sequence similarity over the entire length of protein. The pBLAST search against 7S globulin sequence suggests that *Mc*7S share high similarity with vicilin from pecan (52%), Vic_CAPAN (44%) and SM80.1 (41%). Followed by 7S globulin-1 adzuki bean (36%), β-conglycinin (35%), 8S mungbean storage protein (35%), AraH1 (35%), Korean pine vicilin (33%), phaseolin (33%), and canavalin (33%). The Fig. S4 shows the multiple sequence alignment (MSA) of all these 7S globulins. The MSA showed that the loop I connecting β6-β7 is longer in case of *Mc*7S as compared to other crystal structures. The loop region II and III involved in connecting the N-terminal domain to the C-terminal domain are disordered. The length of loop II is small in *Mc*7S, pecan, *Solanum*, french bean and jackbean. The loop II is longest in peanut, adzuki bean and 8S mungbean. The loop III has almost the same length in all 7S globulins. The loop region IV is near the copper-binding site and the length is longer in *Mc*7S followed by pecan, Korean pine and peanut as compared to other reported crystal structures.

Structural similarity search using Dali Lite indicated that *Mc*7S structure shares the highest similarity with SM80.1, Vic_CAPAN and pecan vicilin; followed by AraH1, Korean pine vicilin, β-conglycinin, 7S globulin-3, 8S mungbean. The least homology is observed with phaseolin and canavalin. All these structures have an overall C-alpha RMSD of less than 2 Å; the slight variation occurs around disorder loop III, loop IV and extended C-terminal helical arm (Fig. 1D). The density for loop II was present in most of the structures except 8S mungbean; while the density of loop III was not observed in most of the 7S globulins. The loop region IV is near the copper-binding site and the density was not observed in any structure. The length of the loop region IV is longer in *Mc*7S as compared to other reported crystal structures. The maximum likelihood (ML) phylogenetic tree classified *Mc*7S with pecan, SM80.1 and Vic_CAPAN, followed by Korean pine vicilin, whereas the second subtree comprised of adzuki bean, canavalin, β-conglycinin, 8S mungbean, phaseolin and AraH1 (Fig. S5).

#### Metal binding site

**Figure 2:**
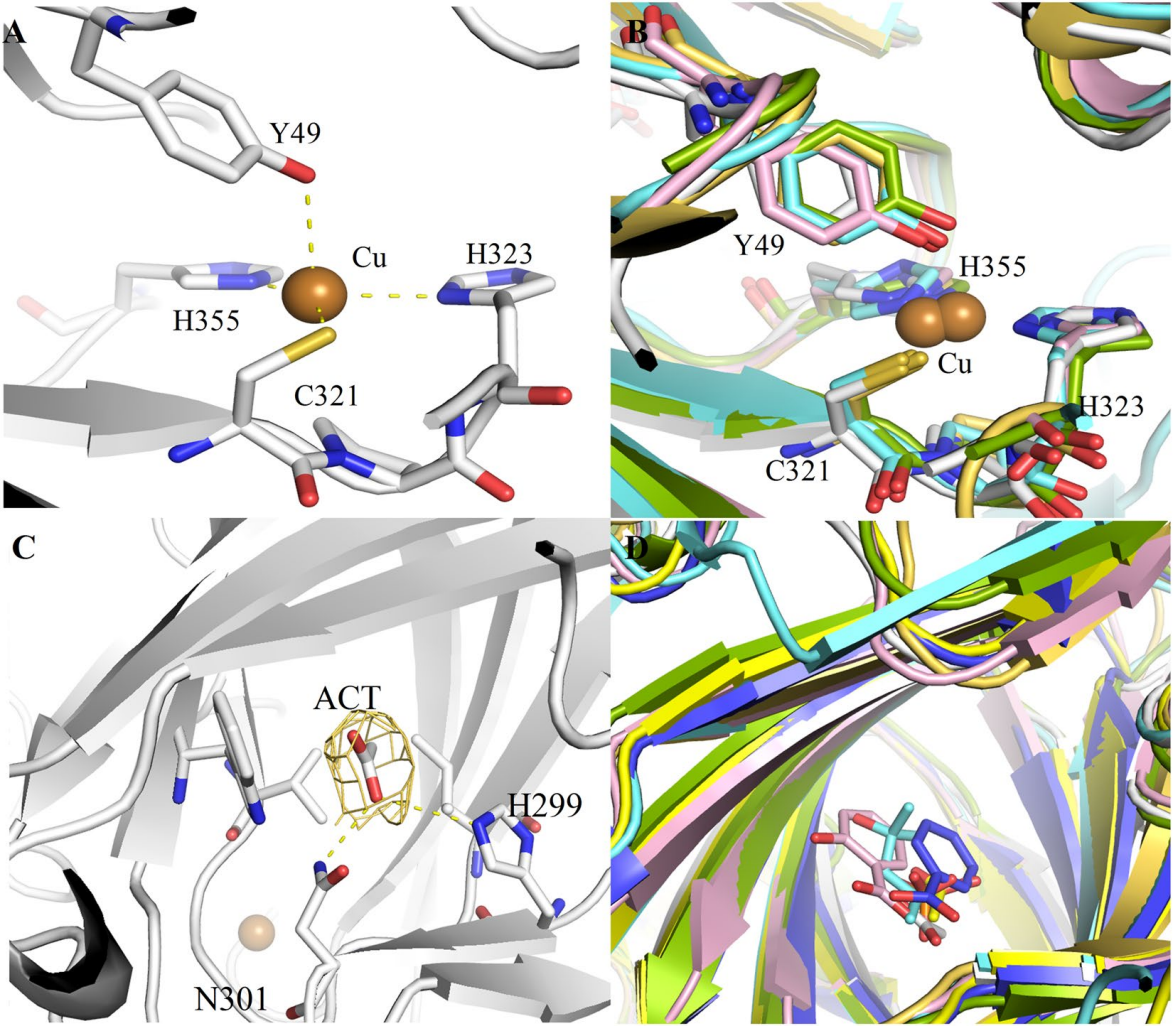
Ligand bound to *Mc*7S. (**A**) Copper (Cu) metal coordination site of *Mc*7S showing trigonal planar geometry. (**B**) Structural superimposition of the metal binding site of *Mc*7S (white), Korean pine vicilin (green), pecan (cyan), *Capsicum annum* Vic_CAPAN (pink) and *Solanum melongena* (golden). (**C**) Interaction of Acetate (ACT) with NE2 atom of His299 and ND2 atom of Asn301 through its O atom. (**D**) Superimposition of acetate (ACT) binding site of *Mc*7S (white) with *Solanum melongena* (golden), Adzuki bean (yellow), Jackbean (purple), Korean pine (green) and pecan (cyan). Residues are labeled as per *Mc*7S.

The *F*o-*F*c map showed the clear electron density near the tyrosine (Tyr49) from the N-terminal domain and the cysteine (Cys321), and two histidines (His323 and His355) from the C-terminal domain. The site was conserved in the phylogenetic subtree members pecan, SM80.1, Vic_CAPAN and Korean pine vicilin. The crystal structure of 7S gobulins from SM80.1, pecan, Vic_CAPAN and Korean pine showed electron density of copper at the site^14–18^. The presence of copper ion in *Mc*7S globulin was further confirmed by inductively coupled plasma mass spectroscopy (ICP-MS) (Table S4**)**. The metal possesses a trigonal planar geometry with copper in the centre as inferred from CheckMyMetal server (Fig. 2A). Structural superimposition suggests that copper-binding site is conserved in pecan (Cys652, His654 and His698), SM80.1 (Cys281, His283 and His325), Vic_CAPAN (Cys417, His419 and His464) and Korean pine vicilin (Cis338, His340 and His379) is shown in Fig. 2B. The copper ion is placed slightly away from the centre of the trigonal plane formed by SG of Cys321, ND1 of His323, and the NE2 of His374. The polder omit map (mFo-Fc) contoured at 2.5 σ for copper ion and coordinating residues are shown in Fig. S6A.

In globulin-3 from adzuki bean, the electron density was observed for a calcium ion, at a different site. The site was not conserved in *Mc*7S globulin. Although, the site was conserved in members of other legume vicilins like canavalin, β-conglycinin, 8S mungbean and AraH1 from *Arachis hypogaea*; no calcium ion-electron density was observed. Thus, the ML phylogeny tree corresponds well with the metal ion binding tendency of vicilins.

#### Acetate binding site

The C-terminal β-barrel domain of *Mc*7S has an evident density for acetate moiety (Fig. 2C). The acetate interacts with NE2 atom of His299 and ND2 atom of Asn301 through its O atom. Superimposition with other 7S globulin shows that the presence of acetate (in SM80.1 and adzuki bean), 2-Methyl-2, 4-pentanediol (MPD) (in pecan), phosphate (in Korean pine), salicylic acid (in Vic_CAPAN) and benzoic acid (canavalin) in the β-barrel of C-terminal domain (Fig. 2D). The acetate binding residues are conserved in most of the 7S globulin (Adzuki bean, β-conglycinin, and AraH1) with few exceptions. In SM80.1, the acetate is stabilized through four hydrogen bonds with Tyr259, Asn261, Arg266 and Lys351. In place of residues Tyr259, Arg266 and Lys351 of SM80.1, the *Mc*7S has His299, Arg381 and Trp306. In Vic_CAPAN, the salicylic acid forms interaction similar to acetate of SM80.1. The carboxy end of salicylic acid interacts with Tyr395, Asn397, Arg402 and Lys490. In canavalin, the carboxyl end of benzoic acid also forms similar interaction as acetate. It interacts with His297, Asn299, Arg376 and Asn284. The Asn284 of canavalin is replaced by Ala286 in *Mc*7S. The MPD also interacts in similar manner, with one end oxygen coordinated by conserved His630, Asn632 and Arg724; whereas the other end oxygen is coordinated by Asn617 (Asn284 of canavalin). In Korean pine, a phosphate ion is found at a similar site. The phosphate coordinates well with Glu383 and conserved Arg405. Therefore, there is a possibility that charge within the pocket can accommodate similar ligands.

### *In-silico* ACE inhibitor prediction

BIOPEP server results showed that *Mc*7S globulin contained many ACE inhibiting peptide but these peptides are inactive within the protein^19^. The human body having many gastrointestinal enzymes and during enzymatic digestion, the protein was cleaved into many fragments^19^. Through *in silico* proteolysis, it was confirmed that ‘VFK’ (PEP) a tripeptide fragment was released on trypsin digestion which has ACE inhibitory activity. The polder omit map (mFo-Fc) contoured at 2.5 σ for VFK residues in *Mc*7S are shown in Fig. S6B. ACE enzyme plays a significant role in blood pressure regulation and its inhibitors are widely used as a drug for the treatment of hypertension, myocardial infarction, heart failure, and diabetic nephropathy^20,21^. The binding affinity of ‘VFK’ peptide was analyzed by docking into the active site of human ACE. The crystal structure of ACE complexed with Lisinopril (PDB ID 1O86) at 2.0 Å was considered for docking of PEP (VFK-peptide) with ACE.

**Figure 3:**
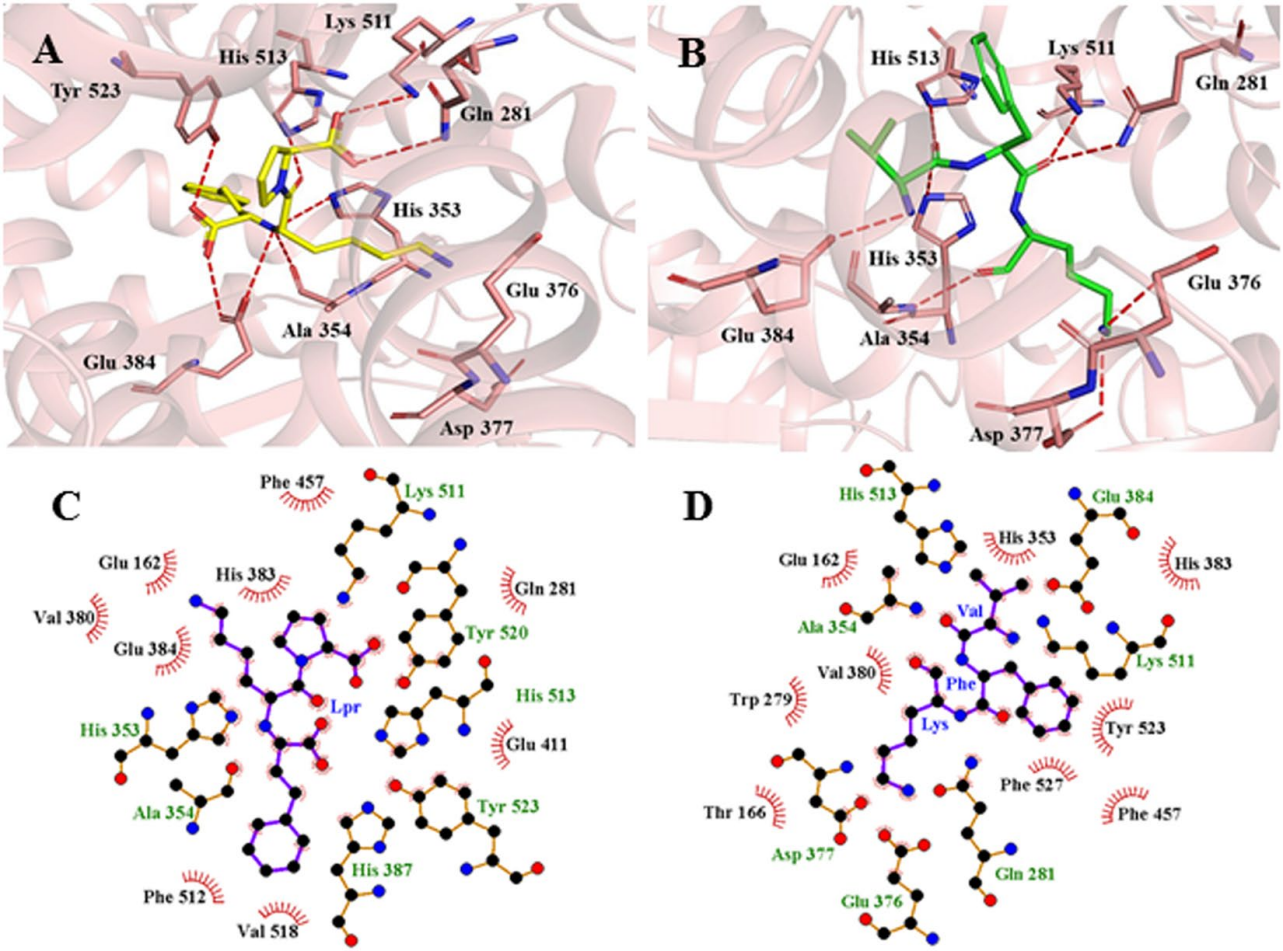
Molecular docking interaction analysis of human angiotensin converting enzyme (ACE) with LPR and PEP. The cartoon representation of ACE with LPR (**A**: yellow color) and PEP (green color). Interacting residues of ACE are shown in stick format in salmon color while red dotted line shows the intermolecular hydrogen bond interactions. 2D representation of interactions of ACE with LPR (**C**) and PEP (**D**) is generated by LIGPLOT.

**Figure 4:**
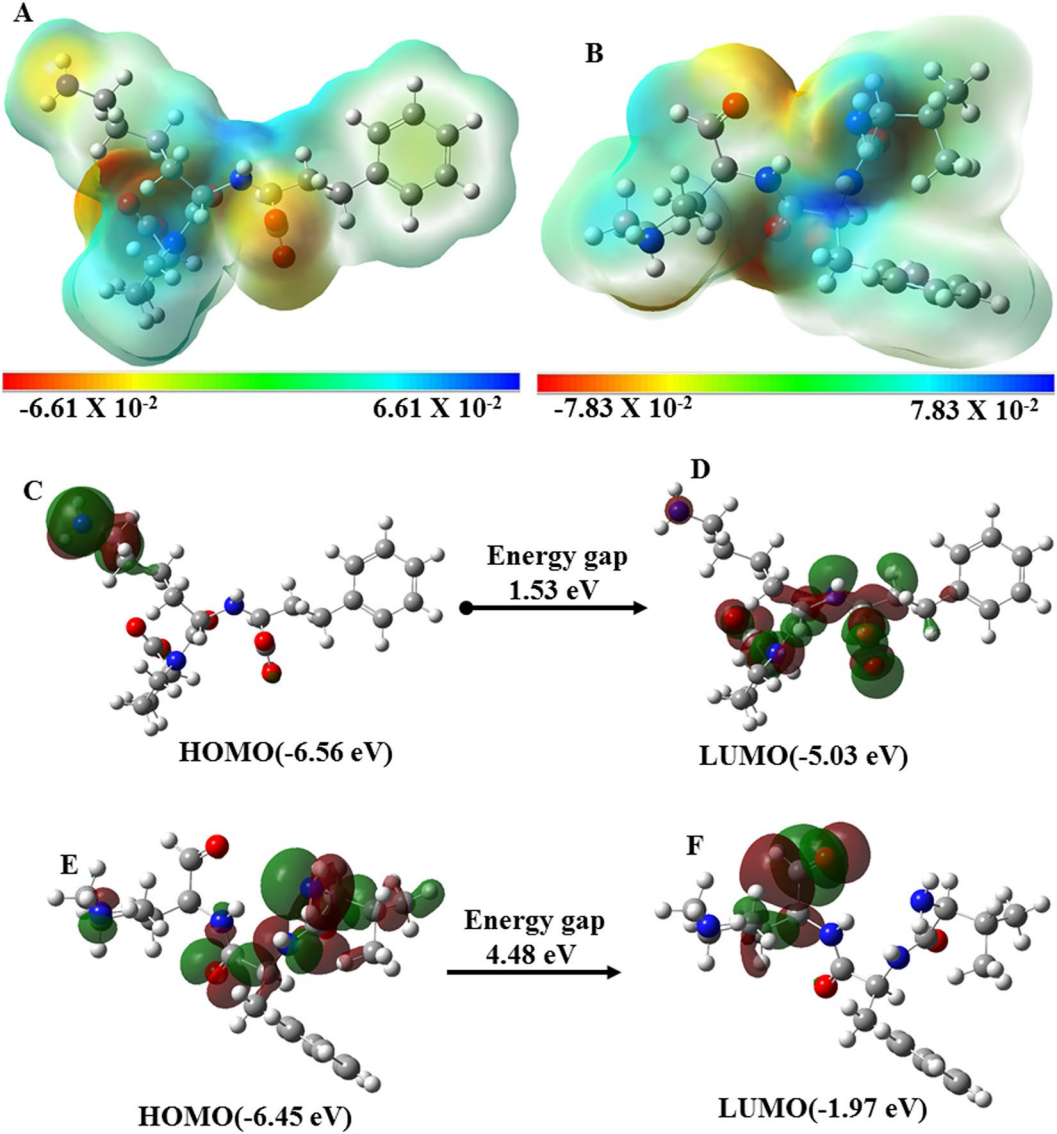
Molecular electrostatic potential (MEP), HOMO and LUMO plots of LPR and PEP generated by DFT/B3LYP method with 6–311G (d,p) basis set in Gaussian 16. MEP plots showing the red (electron rich) and blue (electron poor) regions for: (**A**) LPR and (**B**) PEP. HOMO and LUMO plots of LPR (**C**,**D**) and PEP (**E**,**F**) representing the positive (red) and negative (red) phase distribution in molecular orbital wave function.

Molecular docking of LPR and PEP along with ACE was done using HADDOCK webserver. LPR and PEP show hydrogen bonding interactions with His353, GLu384, Lys511, and His513 as shown in Fig. 3. PEP shows a higher binding affinity (−81.2 +/− 3.8) as compared to LPR (−73.5 +/− 0.5) as shown in Table S5. Density functional theory (DFT), a quantum mechanics approach was used to determine the molecular and electronic properties of LPR and PEP. High and low electron density regions in ligands were calculated by electrostatic potentials as shown in Fig. 4. Highest occupied and lowest unoccupied molecules orbitals (HOMO and LUMO) representation of LPR and PEP are shown in Fig. 4. The higher energy gap of PEP (4.48 eV) as compared to LPR (1.53 eV) indicates that PEP can bind efficiently with ACE as shown in Table S6. Overall molecular docking and quantum mechanics results reveal that PEP can bind more efficiently at the active site of ACE.

### Molecular dynamics

**Figure 5:**
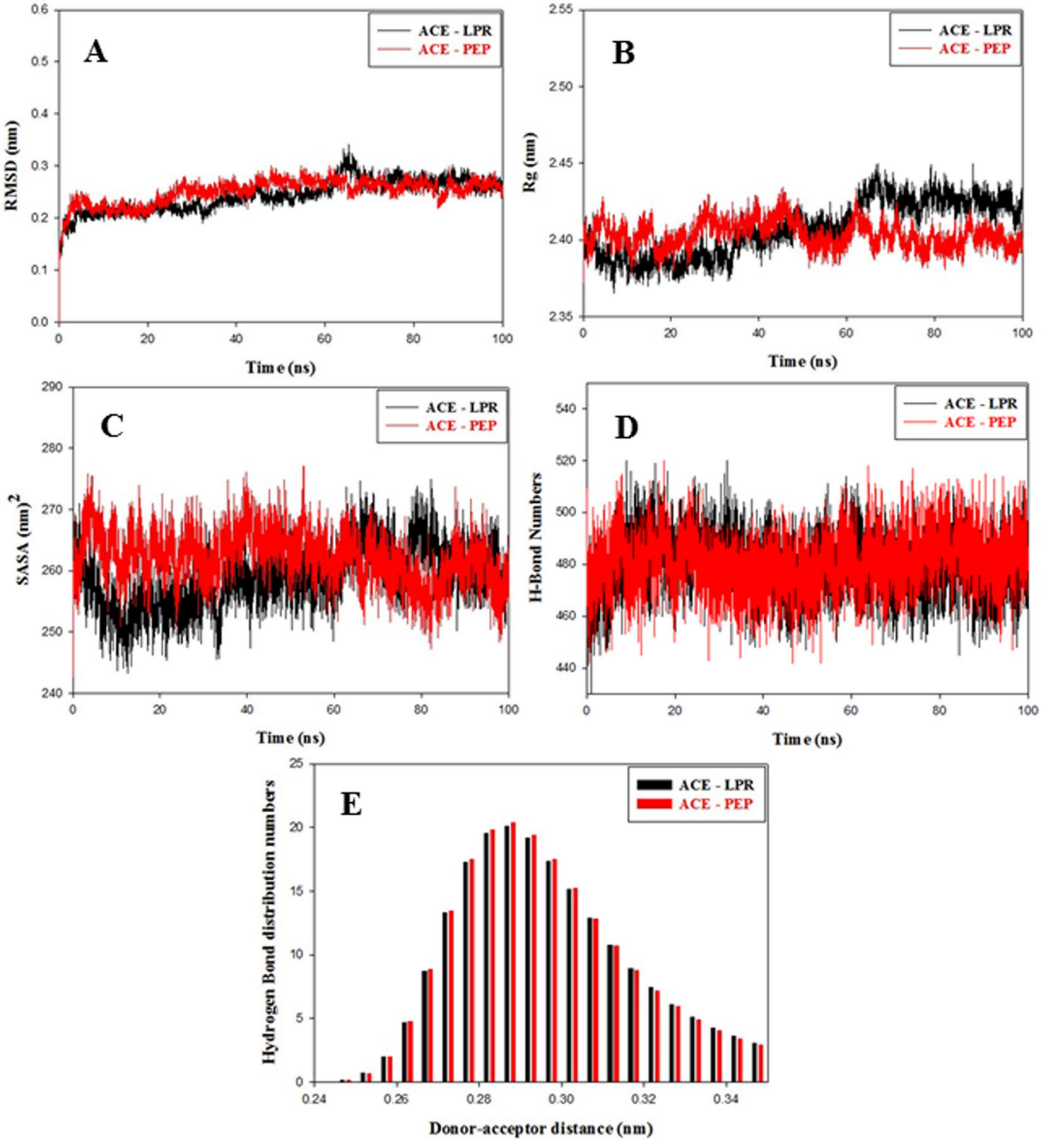
Molecular dynamics results of ACE - LPR (black color) and ACE - PEP (red color) complex during the molecular simulation of 100 ns. (**A**) Root Mean Square Deviation (RMSD), (**B**) Radius of gyration (Rg), (**C**) Solvent Accessible Surface Area (SASA), (**D**) Intra-hydrogen bond numbers and (**E**) Intra hydrogen bond distribution numbers.

Molecular dynamics of ACE-LPR and ACE-PEP complexes were performed using GROMACS 2019.2. Root mean square deviation (RMSD) along Cα backbone atoms were calculated to study the dynamics flexibility of the Cα atoms of ACE-LPR and ACE-PEP complexes. ACE-LPR and ACE-PEP complexes achieved convergence at 65 ns at 0.26 nm and stable during the molecular dynamics of 100 ns as shown in Fig. 5A. Both complexes exhibit an average RMSD of 0.25 nm as shown in Table S7. Radius of gyration (Rg) results indicate that ACE-PEP complex is as stable as ACE-LPR complex as shown in Fig. 5B. Solvent Accessible Surface Area (SASA) was generated to determine the volume of receptor surrounded by solvent molecules during the molecular simulation. SASA results depict that ACE-PEP complex is as compact as ACE-LPR complex as shown in Fig. 5C and Table S7. g_hbond tool of gromacs was used to generate the intra hydrogen bonds in protein-ligand complexes during the molecular dynamics of 100 ns. ACE-PEP complex showed the comparable number of intra hydrogen bonds to ACE-LPR complex as shown in Fig. 5D and Table S7. The distribution of hydrogen bond lengths signifies that ACE-PEP complex form hydrogen bond from high to low affinity which is comparable to ACE-LPR complex as shown in Fig. 5E. Overall, molecular dynamics results confirm that binding of PEP to ACE results in the formation of stable ACE-PEP complex.

### Temperature and pH stability

Scan of far-UV (190–250 nm) CD was used to monitor the secondary structure as well as the conformational stability of *Mc*7S globulin under increasing temperatures (25 to 85 °C) and pH (3 to 11) range (Fig. S7). Spectra showed a small negative peak around 208 nm, a positive peak between 195 nm and 200 nm and a broad negative peak between 212–220 nm which are the characteristic features of protein having predominant β-sheet with little α-helix. It can be concluded that *Mc*7S globulin has predominant antiparallel β-sheet and little α-helix which is comparable with 7S seed storage protein (SSP)s such as phaseolin, canavalin, SM80.1, β-conglycinin^15,22–24^. On changing temperature and pH, very minute change occurs in the range between 195–200 nm, in-conclusion only minor change in the helical region as compared to sheets region. The homo-trimeric state of 7S globulins provides thermo-stability to the protein. The helices from both terminals of each monomer protrude outward to interact with the neighbouring monomers, resulting in the formation of a trimer. It is observed that the electrostatic and hydrophobic interactions stabilize the quaternary structure.

### Antioxidant activity

Antioxidants play a vital role in food processing and storage to reduce the oxidative stress as well as in human health to control the free radicals and oxidative stress-related diseases such as diabetes mellitus, cardiovascular diseases, and neurodegenerative diseases^25,26^. The DPPH radical-scavenging activity was carried out to find the antioxidant activity of the *Mc*7S protein. DPPH is a synthetic, stable free-radical in aqueous or ethanol and has been widely used to assess the radical scavenging ability of various samples. By accepting an electron or hydrogen radical, DPPH becomes a stable diamagnetic molecule and change in the intensity of purple colour of free DPPH radical was measured at 517 nm. The DPPH scavenging activity increased reliably with the change in concentration of *Mc*7S globulin from 0.0–1.5 mg/ml, while the scavenging activity of control compound Ascorbic acid reached a maximum plateau from 0.5 to 1.0 mg/ml (Fig. S8). Thus, results showed that *Mc*7S has antioxidant activity. Additionally, the basic fold of seed storage 7S globulins has evolved from plant germin, an Mn-binding protein with oxalate oxidase and superoxide dismutase activities^27^.

### Glycosylation assay

Glycosylation governs the stability of the protein and provide resistance against denaturation. It plays an important role in protein folding, stability, and interaction as well as in protein trafficking. The glycosylation changes the net charge on the protein surface and largely affects the emulsifying ability and emulsion stability. The 7S globulin pre-pro-proteins are synthesized in the endoplasmic reticulum, and glycosylated in-order to be transported to the vacuoles for storage. Molisch’s test, a sensitive chemical test for detection of carbohydrate moiety was utilized to detect the glycosylation property of *Mc*7S. By the addition of *Mc*7S globulin, a purple coloured ring appeared at the junction of two liquids, which indicates that the purified protein has a carbohydrate moiety (Fig. S9). The N-glycosylated in native β-conglycinin and phaseolin is at Asn328 and Asn262, respectively. Sequence and structural superimposition show that Asn residue is absent from both the position in *Mc*7S, which suggests that N-glycosylation site is different from phaseolin and β-conglycinin. The N-glycosylation occurs on Asparagine residue occurring in the Asn-X-Ser/Thr stretch where X can be any amino acid. This stretch was observed at residue’s position Asn255-Gln256-Thr257 in *Mc*7S. Therefore, the probable N-glycosylation site in *Mc*7S can be Asn255.

## Discussion

The *Mc* plant has several nutritional and nutraceutical values. The SDS-PAGE profile of the seed extract suggested the presence of a ~52 kDa in major amount in *Mc*. The MALDI-ESI-MSidentified fragments suggested the protein to be homologous to 7S globulin. The protein sequence was derived by aligning contig sequences deposited in the Short Read Archive (SRA) database. Three-dimensional structure of *Mc*7S globulin revealed that it has predominantly antiparallel β-sheets as compare to alpha-helix, similar to that of other 7S globulins. The trimer formation around the three-fold crystallographic axis of *Mc*7S resembles that of the other 7S globulins. Literature suggests the trimeric arrangement limits the proteolysis and provides thermal stability^28,29^. Although the sequence homology of Korean pine with *Mc*7S is less, the ML phylogeny tree classified them together. Moreover, a similar copper-binding site was observed in Korean pine along with pecan, SM80.1 and Vic_CAPAN. As there was no copper ion added during the purification or crystallization trials, the presence of copper ion in *Mc*7S globulin was the property of the protein.

To determine the active biopeptide *Mc*7S, *in silico* study was performed and VFK (PEP) a tripeptide fragment was released by trypsin digestion. Molecular docking shows that PEP possesses a higher binding affinity for ACE as compared to LPR. Density functional theory analysis reveals that PEP is more reactive than LPR. Molecular dynamics simulations results confirm that binding of PEP to ACE results in formations of stable ACE-PEP complex. Several molecular docking and simulation studies were used to predict the efficiency of binding of the ligand with macromolecules^30–35^. The ACE inhibitors such as captopril, lisinopril, enalapril, ramipril and perindopril have shown common side effects such as headache, dizziness, cough, low blood pressure, hypotension, nausea, and angioedema^36–38^. Since *Momordica charantia* is considered as a novel plant with diverse applications in food industry as well as in the therapy of many diseases such as diabetes and atherosclerosis. Identification of ACE inhibiting peptide fragments from this plant will show better activity with lower side effects^39^. Variations in secondary structures of *Mc*7S at different pH and temperatures were conducted using Circular dichroism. The *Mc*7S globulin is very stable at different pH and temperature; and probably has a high denaturing temperature similar to 7S globulin from soy protein^40^. Therefore, it is suitable for an application in the food industry where products are needed to maintain under higher temperatures during processing.

The *Mc*7S has radical scavenging ability, as suggested by DPPH assay. The superoxide dismutase radical scavenging ability was observed in Vic_CAPAN, the 7S globulin from *Capsicum annum*^17^. The antioxidants play an important role, including defending against oxidative damage in the major signaling pathways of cells in the biological system. Although several commercially available synthetic antioxidants are available, their usage is restricted due to their toxic side effects^41^. Thus, efforts need to made to identify and development of alternative natural antioxidants. The common oxidation products are formed from sulphur-containing (cysteine and methionine), aromatic (tryptophan, tyrosine, and phenylalanine) and imidazole-containing (histidine) residues compared to aliphatic residues. The amino acid composition of these residues in *Mc*7S is similar to SM80.1 and Vic_CAPAN; therefore, they may share functional properties too. Other factors that are responsible for the antioxidant ability of the protein are its amino acid composition, pH, its source, glycosylation, digestion, and absorption^42^. The 7S globulin from *Momordica charantia* is a glycoprotein similar to 7S globulin from *Pisum sativum*^43^, β-conglycinin from *Glycine max*^44^, and phaseolin from *Phaseolus vulgaris*^45^. The glycosylation increases the stability and solubility of the protein.

In conclusion, the *Mc*7S protein was crystallized and characterized by using computational and *in-vivo* study. Crystal structure of *Mc*7S reveals that it is trimer and consists of antiparallel β-sheets. ICP-MS confirmed the presence of copper ions coordinated with Cys321, His323, and His374 of *Mc*7S. *In silico* analysis indicate the release of tripeptide (VFK) from protein during digestion by digestive enzymes in humans. Further, molecular docking, quantum mechanics and molecular dynamics confirm that PEP binds with ACE with higher binding affinity than LPR and tends to form stable ACE-PEP complex. Already reported ACE inhibitors such as captopril, lisinopril, enalapril, ramipril and perindopril have several side effects, so usage of PEP might result in lesser side effects. Secondary structure content of *Mc*7S sustains upto 85 °C which indicates to use it at high temperatures in food industry. Moreover, *Mc*7S globulin has antioxidant property which plays a significant role in the treatment of different diseases. Glycosylation property of *Ms*7S globulin plays role in protein stability and in antioxidant activity. This study suggesting that *Mc*7S protein and its peptide could be used as a natural source of antioxidant and as a raw material, respectively in food industry and human health system to reduce the oxidative stress and free radicals.

## Methods

### Purification of *Mc*7S globulin

The seeds of *Mc* were purchased from local market. The seed coats were removed and decorticated seeds were soaked overnight in buffer A (50 mM Tris Buffer pH 7.4). Soaked seeds were flash-frozen with liquid nitrogen and homogenized by mortar and pestle in buffer A. The crude extract was subjected to centrifugation at 12000 rpm, 4 °C for 2 h to obtain a clear supernatant. The supernatant was retrieved after careful removal of lipid layer. The clear supernatant was subjected to ammonium sulphate precipitation step in which different percentage of ammonium sulphate (0–20%, 20–40%, 40–60%, 60–80% and 80–100%) was slowly added while stirring at 4 °C. Eluted fractions were analysed on 12% SDS-PAGE and fractions containing 7S globulin were dialyzed thrice in 1 L of buffer A for 12 hrs at 4 °C. The dialyzed sample was applied onto Affi-Gel Blue matrix (Bio-Rad Laboratories, Hercules, California, USA) pre-equilibrated with buffer A. The column was washed with buffer A and bound proteins were eluted using a gradient of NaCl (0.1, 0.2, 0.3, 0.4, 0.5 and 1.0 M) in buffer A. Eluted fractions were examined on 12% SDS–PAGE and the fractions with 7S globulin were pooled and concentrated to 10 mg/ml using Amicon Ultra-15 (10 000 Da MWCO, Millipore). Further, size exclusion chromatography was performed for final step purification. The concentrated protein fraction was loaded on HiLoad 16/60 Superdex 200 pg column pre-equilibrated with buffer A and 150 mM NaCl, at a flow rate of 0.5 ml/min on the ÄKTA purifier (GE Healthcare). By measuring the absorbance at 280 nm, the protein elution profile was monitored and analysed on 12% SDS-PAGE under reducing condition. Peak containing 7S globulin eluted at 69.2 ml, which correspond nearly to the predicted molecular weight of 7S globulin trimers. The result was validated by calibrating the column with high molecular weight standards.

### Partial internal sequencing and gene sequence deduction from contigs

The purified protein was electrophoresed on 12% SDS-PAGE and the protein bands were excised and partially digested by Trypsin. The partially digested fragments were utilized for Matrix assisted laser desorption ionization electrospray ionization mass spectrometry (MALDI-ESI-MS) followed by reverse phase separation. The data obtained were analysed by the MASCOT search engine. The experiment was carried out at protein sequencing facility at National Botanical Research Institute, Lucknow, India. The obtained peptide fragments were searched in ESTs database, however, no hits were obtained. Therefore, the contig sequence data related to bitter melon (*Momordica charantia*) that has been deposited in the GenBank Short Read Archive (SRA) with the accession number SRP004091 was downloaded. The accession numbers for the individual experiments of normalized sequence data is SRX030203^46^. The composition of cDNA transcripts analysed from this experiment was found to encode cupin 2, PV100 a seed storage protein. The sequence read archive nucleotide BLAST was utilized to identify these contig sequences using cupin 2, PV100 (gi|3808062) as query. DNASTAR Lasergene Molecular Biology Suite was used for assembling contigs. The gene sequence was converted to protein sequence using ExPASy translate tool (https://web.expasy.org/translate/).

### Crystallization, data collection, data processing, structure determinmixture contained protein samplesation and refinement

The purified *Mc*7S globulin was crystallized by the sitting drop vapour-diffusion method in 96-well crystallization plates (Hampton Research) at 293 K. The 10 mg/ml protein concentration was used for setting crystal trays. Initial hits were obtained in crystal screen (Hampton Research, USA). The diffraction quality hexagonal plate-like crystals were obtained in 0.1 M sodium acetate trihydrate pH 4.6 and 2 M sodium chloride within 2–3 days. Crystal was soaked in a cryoprotectant solution containing reservoir solution along with 30% glycerol followed by flash freezing in liquid nitrogen prior to diffraction. Diffraction data were collected using a Bruker Microstar rotating anode X-ray generator (CuKα wavelength = 1.54 Å) and MAR345dtb image plate detector, positioned at 270 mm distance from crystal at 100 K temperature. X-ray exposure of 10 min and 1° oscillation per frame was used for collection of 100 diffraction images. The crystal diffracted to a resolution of 3.1 Å at the home source. HKL2000 was used for indexing, integrating and scaling the data^47^. The structure of *Mc*7S globulin was solved by molecular replacement method using vicilin from pecan (*Carya illinoinensis*) (PDB ID: 5E1R) as template^48^. The transformed coordinates were refined with phenix.refine from Phenix^49^. The manual model building was carried out with graphics programs COOT^50^. The models were evaluated using the program Molprobity^51^. Metal geometry was checked using CheckMyMetal web server^52^. The figures were generated using the program PyMOL and ESPript program^53,54^.

### Metal analysis using inductively coupled plasma mass spectroscopy (ICP-MS)

ICP-MS of the *Mc*7S protein sample was performed to detect the presence of copper metal ion content in the purified protein. The sample was prepared using a previously described method^55^. The protein was concentrated using Amicon Centricon-10 concentrator up to ∼10 mg/ml, further, the acid mineralization was done to solubilize the organic content which may otherwise precipitate during experimentation and lead to choking of the tubing system in the instrument. For this, an equal volume of an ultrapure analytical grade of HCl was mixed with ultrapure analytical grade HNO_3_ in a ratio 1:1. This mixture was added to an equal volume of protein sample and heated in a dry bath for 2 h at 100 °C. The sample volume was brought to 5 ml using doubly deionized water. Liquid calibration standards (10 ppb, 100 ppb and 1000 ppb) were prepared for constructing a calibration curve. Signal intensities of the unknown samples were compared to the calibration curve and the intensity of the analyte was determined.

### *In silico* ACE inhibitor prediction

The *Mc*7S protein sequence was evaluated for the presence of peptide fragments using BIOPEP server (http://www.uwm.edu.pl/biochemia/index.php/en/biopep). Further, Expasy peptide cutter server was utilized to check the digestion site in *Mc*7S. ‘Valine Phenylalanine Lysine-VFK’ a tri-peptide fragment confirmed by BIOPEP server was considered for molecular docking using HADDOCK (High Ambiguity Driven protein-protein DOcking)^56,57^. Best generated conformations were analysed and visualized in PyMOL^53^. Interaction figures were made in Pymol and LIGPLOT^53,58^. Density functional theory (DFT) studies were performed using DFT/B3LYP method with 6–311G (d,p) basis set in Gaussian 16^59–62^. The details of molecular docking and density functional theory is mentioned in Supplementary information.

### Molecular dynamics

Molecular dynamics was done to determine the dynamic stability of ACE-LPR and ACE-PEP complexes^20^. Crystal structure of *Mc*7S and human angiotensin converting enzyme in complex with Lisinopril (PDB ID:1O86) were considered as a starting point for the molecular simulation. Molecular dynamics was performed using GROMOS 54a7 force field in GROMACS 2019.2 suite on an Ubuntu-based workstation^63,64^. LPR topology files were produced by PRODRG and partial atomic charges were calculated using DFT/B3LYP method with 6–311G (d,p) basis set in Gaussian 16^59–62,65^. Long-range electrostatics were measured using Particle Mesh Ewald (PME) and coulomb interactions were determined within a cut off the radius of 12 Å^66^. The final molecular dynamics was run for 100 ns. The molecular dynamics simulation study described in detail in Supplementary information.

### Circular dichroism studies

Secondary structure and conformational stability of protein were analysed by using Jasco J-1500 CD spectrometer equipped with a Peltier thermostat cell holder (PTC-510) temperature controller. Far-UV (190–250 nm) CD spectra were recorded at a protein concentration of 0.2 mg/ml in sodium phosphate buffer (20 mM, pH 7.4) in 1 mm quartz cell at 25 °C. The conformational stability of protein was examined by recording the spectra at different temperature (25–85 °C) and at different pH (3–11) in triplicates, and then averaged the data for analysis. The CD spectra were analyzed and intensities were expressed as mdeg.

### DPPH radical-scavenging activity

The percent of DPPH radical scavenging activity was determined according to the Zheng *et al*. with some modification^67^. The reaction mixture contains 1 ml of 0.1 mM DPPH solution in ethanol with 1 ml of protein sample in 50 mM Tris buffer pH 7.4 at different concentrations (62.5–1500 µg/ml) and incubated in the dark for 1 hr at room temperature. Using UV-Vis spectrophotometer, the color shifts were read at 517 nm. Ascorbic acid was used as positive control with different concentrations. The scavenging activity was expressed as follows:

$$ \% \,scavenging\,activity=\frac{Ac-As}{Ac}\ast 100$$

where *As* was the absorbance of mixture contained protein samples and *Ac* was the absorbance of control (only DPPH solution).

### Glycosylation assay

The glycosylated nature of the purified protein was confirmed by Molisch Test^68^. Briefly, 1 ml of protein sample (0.1 mg/ml) in buffer A (50 mM Tris Buffer pH 7.4) was placed in a test tube; 1–2 drops of Molisch’s reagent (10% α-naphthol in ethanol) were added and mixed gently. Along the side of the inclined tube, 1–2 ml of concentrated sulphuric acid was slowly added and the same was done with the negative control (only buffer instead of protein) as well as with positive control (glucose solution).

#### Protein data bank submission

The atomic coordinates and structure factors of *Momordica charantia* 7S globulin has been deposited with accession code 6KM8.

## Supplementary information

Supplementary file.
