# Supplementary material for: Structural characterization and in-silico analysis of Momordica charantia 7S globulin for stability and ACE inhibition
Source: Sci Rep. 2020 Jan 24;10:1160. doi: 10.1038/s41598-020-58138-9 (PMC6981215; doi:10.1038/s41598-020-58138-9)
Supplement: Supplementary file 1 — Supplementary file. [file 41598_2020_58138_MOESM1_ESM.docx]

**Structural characterization and *in-silico* analysis of *Momordica charantia* 7S globulin for stability and ACE inhibition**

**Pooja Kesari^a#^, Shivendra Pratap^a#^, Poonam Dhankhar^a#^, Vikram Dalal^a^, Manisha Mishra^b^, Pradyumna Kumar Singh^b^, Harsh Chauhan^a^, Pravindra Kumar^a*^.**

^a^Department of Biotechnology, Indian Institute of Technology Roorkee, Roorkee, Uttarakhand-247667, India

**^b^**Plant Molecular Biology Division, Council of Scientific and Industrial Research (CSIR)-National Botanical Research Institute, Lucknow. India.

*Corresponding Author (Email Id: [pravinmcu@gmail.com](mailto:pravinmcu@gmail.com))

**^#^** Equal contribution

**Methods**

***In silico* ACE inhibitor prediction**

The *Mc*7S protein sequence was evaluated for the presence of peptide fragments using BIOPEP server (<http://www.uwm.edu.pl/biochemia/index.php/en/biopep>). A number of Angiotensin converting enzyme (ACE) inhibiting peptide fragments were released from *Mc*7S globulin protein and further Trypsin digestion site was evaluated using Expasy peptide cutter (<https://web.expasy.org/peptide_cutter/>). The *in-silico* proteolysis results showed ‘Valine Phenylalanine Lysine-VFK’ a tri-peptide fragment having ACE inhibition properties confirmed by BIOPEP server was selected for further docking studies. For molecular docking simulation, the atomic coordinates of the protein (ACE) were retrieved from human angiotensin converting enzyme in complex with Lisinopril (PDB ID:1O86) and Lisinopril (LPR) and PEP (VFK-peptide) were used as ligands ^1^. The molecular docking was performed using HADDOCK (High Ambiguity Driven protein-protein DOcking) and the important interacting residues were mentioned as active residues ^2,3^. HADDOCK generated the 190 structures in 7 clusters, which represents the 95.0% of water refined model generated. Best generated conformations were analysed and visualized in PyMOL ^4^. Interaction figures were made in PyMOL and LIGPLOT ^4,5^. Density functional theory (DFT) studies were performed using DFT/B3LYP method with 6-311G (d,p) basis set in Gaussian 16 ^6-9^. DFT was used to generate polarity of ligands and nature of highest occupied and lowest unoccupied molecules orbitals (HOMO and LUMO) to determine the nucleophilic and electrophilic activity.

**Molecular dynamics**

Molecular dynamics was performed to determine the dynamic stability of ACE-LPR and ACE-PEP complexes ^1^. The coordinated from crystal structure of Mc7S and human angiotensin converting enzyme in complex with Lisinopril (PDB ID:1O86) were used as a starting point for the molecular simulation. Molecular dynamics was done using GROMOS 54a7 force field in GROMACS 2019.2 suite on an Ubuntu-based workstation ^10,11^. LPR topology files were generated by PRODRG and partial atomic charges were calculated using DFT/B3LYP method with 6-311 G (d,p) basis set in Gaussian 16 ^6-9^ ^12^. The protein structures were inserted into a triclinic box of volume of 669.44 nm^3^ with a 10 Å distance from the nearest atom of protein. The system was neutralized by addition of counter-ions (Na^+^) using genion tool. The system was energy minimized using steepest descent algorithm for 50000 steps to remove the steric clashes. Two steps equilibration steps: constant number of particles, volume, and temperature (NVT) and a constant number of particles, pressure, and temperature (NPT) were performed at 300K for 1 ns and coordinates were generated at every 1 ps. Long-range electrostatics were measured using Particle Mesh Ewald (PME) and coulomb interactions were determined within a cut off the radius of 12 Å ^13^. The final molecular dynamics was run for 100 ns for the said respective temperatures. Molecular dynamics simulation trajectories were used to generate the Root Mean Square Deviation (RMSD), Radius of gyration, Solvent Accessible Surface Area (SASA), number of hydrogen bonds results and principal component analysis (PCA).

**Supplementary Data D1.** Gene sequence deduction from contigs. The contig sequence data related to bitter melon (*Momordica charantia*) has been deposited in the GenBank Short Read Archive (SRA) with the accession number SRP004091. The accession numbers for the individual experiments of normalized sequence data is SRX030203. The composition of cDNA transcripts analysed from this experiment was found to encode cupin 2, PV100 a seed storage proteins. The sequence read archive nucleotide BLAST was utilized to identify these contig sequences using cupin 2, PV100 (gi|3808062) as query. DNASTAR Lasergene Molecular Biology Suite was used for assembling contigs.

AB019195.1:1130-2451 AGCCGTGTAAATCAAGTAGCCATTCGACGAACAGA---GCAAGAACAGAGCAACAACCCC

reverse agccgcggcaaccaggtggaaattccgcgcgaagaacaggaacagggccgccataacccg

118981.2 ------------------------------------------------------------

100733.2 ------------------------------------------------------------

51475.2 ------------------------------------------------------------

140410.2 ------------------------------------------------------------

246005.2 ------------------------------------------------------------

227217.2 ------------------------------------------------------------

11427.2 ------------------------------------------------------------

72791.2 ------------------------------------------------------------

236654.2 ------------------------------------------------------------

236612.2 ------------------------------------------------------------

105306.2 ------------------------------------------------------------

AB019195.1:1130-2451 TACTACTTTCAGGAACAGCGTTTTCAATCAAGGTACAGGTCTGACGAGGGCCATTGGAGG

reverse tattattttcatgaacatagctttcagagccgctttcgcagcgaagatggccattggcgc

118981.2 ------------------------------------------------------------

100733.2 ------------------------------------------------GGCCCCTGCCGG

51475.2 ------------------------------------------------------------

140410.2 ------------------------------------------------------------

246005.2 ------------------------------------------------------------

227217.2 ------------------------------------------------------------

11427.2 ----------------------------------------------------------AG

72791.2 ----------------------------------------------------------GG

236654.2 ------------------------------------------------------------

236612.2 ------------------------------------------------------------

105306.2 ------------------------------------------------------------

AB019195.1:1130-2451 GTGCTGGAGAGATTCTCCGAGAGGTCGGAGCTTTTGAAAGGAATTAAAAACCAGCGATTG

reverse gtgctggaacgctttagccagcgcagcgatgtgctgcgcggcattgaaaaccagcgcttt

118981.2 ------------------------------------------------------------

100733.2 GATCACCATGACATCTCCACG-----------------------------------TTCA

51475.2 ------------------------------------------------------------

140410.2 ------------------------------------------------------------

246005.2 ------------------------------------------------------------

227217.2 ------------------------------------------------------------

11427.2 ATGAGGGCACGC------------------------------------------------

72791.2 CTTTTGAAAATCTCATCCGC----------------------------------------

236654.2 ------------------------------------------------------------

236612.2 ------------------------------------------------------------

105306.2 ------------------------------------------------------------

AB019195.1:1130-2451 GCACTTCTTGAGGCCCGCCCTCACACCTTCATCGTCCCCCATCACTTGGATGCGGAATGT

reverse gcgattctggaagcgcgcccgcagacctttattattccgcatcatctggatgcggaaacc

118981.2 ------------------------------------------------------------

100733.2 GCG--------------TTATAAGACTCTCTTTTTGTTTCCTTCCTTTCTTGAAC-AACC

51475.2 ---------------------TATGATTTTTTCCTCGCCTCTCACTGT------------

140410.2 ----------------------TATGATTTTTCCTCGCCTCTCACTGT------------

246005.2 ------------------------------------------------------------

227217.2 ------------------------------------------------------------

11427.2 ------------------------------------------------------------

72791.2 ------------------------------------TTCCTTTCCTTTTATGTTGAATGT

236654.2 ------------------------------------------------------------

236612.2 ------------------------------------------------------------

105306.2 ------------------------------------------------------------

AB019195.1:1130-2451 GTTCTCT----------TGGTCGTAAGAGGAAGAGCGACGATCA-CTACAGTAGTGCAGG

reverse gtgctgc----------tggtggtgcgcggccgcgcggcgatta-ccaccgtggtgcagg

118981.2 ------------GCCCTTCCTCTTACAACCAAGAGAACTGTTTCAGCGTCCAAGTGGTGG

100733.2 GTAGTTATGGCCGCCCTTCCTCTTACAACCAAGAGAACCGTTTCAGCGTCCAAGTGGTGG

51475.2 ------------------------------------------------------------

140410.2 ------------------------------------------------------------

246005.2 ------------------------------------------------------------

227217.2 ------------------------------------------------------------

11427.2 ------------------------------------------------------------

72791.2 GAGTTC------------------------------------------------------

236654.2 ------------------------------------------------------------

236612.2 ------------------------------------------------------------

105306.2 ------------------------------------------------------------

AB019195.1:1130-2451 AGAAGAGGGAAACTAGGAAAGAGAGTTACAACGTTGAATCAGGGGATGTTATGACGATCC

reverse aacgcaaagaaaccaaacgcgaaagctataacgcggaacgcggcgatgtgatggtgattc

118981.2 GGGATGATGAAGGTCTGGGGGCGGGCCTCAAGTATGGCAAATCGGTTGTTTT--CAATTC

100733.2 GGGATGGTGAAGGTCTGAGGGCGGGCCTCAGGTATGGCAAATCGGTTGTTTT--CAATTC

51475.2 ------------------------------------------------------------

140410.2 ------------------------------------------------------------

246005.2 ------------------------------------------------------------

227217.2 ------------------------------------------------------------

11427.2 -------------------------------------------------C----------

72791.2 ------------------------------------------------------------

236654.2 ------------------------------------------------------------

236612.2 ------------------------------------------------------------

105306.2 ------------------------------------------------------------

AB019195.1:1130-2451 CGGCCGGAACAACTTTATACTTGGCAAACCAAGAAAATGAAGATCTCCAGATCGTGAAAT

reverse cggcgggcgcgaccatttatctggtgaaccatgaaaacgaagatctgcagattgtgaaac

118981.2 CTCT-------------TAGAACGTCAGACCTCTGTGAGAACCTCTCCAGCACCCTCCAA

100733.2 CTCT-------------TAGAACGTCAGACCTCTGTGAGAACCTCTCCAGCACCCTCCAA

51475.2 ------------------------------------------------------------

140410.2 ------------------------------------------------------------

246005.2 ------------------------------------------------------------

227217.2 ------------------------------------------------------------

11427.2 ------------------------------------------------------------

72791.2 ------------------------------------------------------------

236654.2 ------------------------------------------------------------

236612.2 ------------------------------------------------------------

105306.2 ------------------------------------------------------------

AB019195.1:1130-2451 TGGTTCAACCCGTCAACAATCCGGGCGAATTCAAGGATTATCTATCCGCCGGAGGTGAAT

reverse tgattcagccgattaacaacccgggcgaatttaaagattatctgagcgcgggcggcgaag

118981.2 TGGC------CATCCTCAGACCTGAACC----------------TTGATTGGAAACTATG

100733.2 TGGC------CATCCTCAGACCTGAACC----------------TTGATTGGAAA-----

51475.2 ------------------------------------------------------------

140410.2 ------------------------------------------------------------

246005.2 ------------------------------------------------------------

227217.2 ------------------------------------------------------------

11427.2 ------------------------------------------------------------

72791.2 -----------CTTCGCTTCTTGGTCTACTTCGTTC------------------------

236654.2 ------------------------------------------------------------

236612.2 ------------------------------------------------------------

105306.2 ------------------------------------------------------------

AB019195.1:1130-2451 CTCAAGCATATTACAGCGTTTTCAGCAATGATGTTCTCGAAGCTGCTCTAAACATTCCAC

reverse atcagagctattataccgtgtttagcaacgatgtgctggaagcggcgctggatattccgc

118981.2 CTCATGGAAGTAGTAGGGATTGTGCCTGCCCTGTTCTTGCTCCTCTCGGGGGATTTCCAC

100733.2 ------------------------------------------------------------

51475.2 -----------------------------GCTGCTTGAAAACCCTCTCTAGCCTATCACG

140410.2 -----------------------------GCTGCTTGAAAACCCTCTCTAGCCTATCACG

246005.2 ------------------------------------------------------------

227217.2 ------------------------------------------------------------

11427.2 ------------------------------------------------------------

72791.2 ---------------------------ATTATGTTCTCTTTCCCGGC-------------

236654.2 ------------------------------------------------------------

236612.2 ------------------------------------------------------------

105306.2 ------------------------------------------------------------

AB019195.1:1130-2451 GGGATAAACTAGAGAGGATATTCAAGCAGAGAAGGGAGAGAGGAGGAAAAATCGTAAGGG

reverse gcgatcgcctggaacgcgtgtttaaacagcatagcgaacgccgcggcaaaattattaaag

118981.2 TTGATT------------------------------------------------------

100733.2 ------------------------------------------------------------

51475.2 TGGGAT------------------------------------------------------

140410.2 TGGGAT------------------------------------------------------

246005.2 -------------------------GCAGCACAGTGAGAGGCGAGGAAAAATCATAAAGG

227217.2 ------------------------------------------------------------

11427.2 ------------------------------------------------------------

72791.2 ---------TAGGAAGGTT-----------------------------------------

236654.2 ------------------------------------------------------------

236612.2 ------------------------------------------------------------

105306.2 ------------------------------------------------------------

AB019195.1:1130-2451 CATCACAAGAGCAACTAAGAGCGTTGAGCCAACGAGCCACCTCCGTAAGAAAAGGTAGTC

reverse gcagccaggaacagctgaaagcgctgagccagcgcgcgaccagcgtgcgcaaaggcggcc

118981.2 ------------------------------------------------------------

100733.2 ------------------------------------------------------------

51475.2 ------------------------------------------------------------

140410.2 ------------------------------------------------------------

246005.2 GTTCACAAGAGCAGCTGAAAGCGTTGAGCCAACGCGCCACTTCTGTCAGAAAAGGTGGCC

227217.2 ----------------------------------------TTCTGTCAGAAAAGGTGGCC

11427.2 ------------------------ATCTCGAAGGAGCCGGCTCCTTCTGCAACGAATACC

72791.2 ------------------------------------------------------------

236654.2 ------------------------------------------------------------

236612.2 ------------------------------------------------------------

105306.2 ------------------------------------------------------------

AB019195.1:1130-2451 GGGGAGTCAGAGCTCCGATCAAGCTCGAAAGCCAGACCCCTGTTTACAACAACCAATACG

reverse gcggcacccgcgcgctgattaaactggaaaaccagaccccggtgtatagcaaccagtatg

118981.2 ------------------------------------------------------------

100733.2 ------------------------------------------------------------

51475.2 ------------------------------------------------------------

140410.2 ------------------------------------------------------------

246005.2 GAGGAACCCGAGCTCTAATCAAGCTCGAAAACCAAACCCCTGTTTACAGCAACCAATACG

227217.2 GAGGAACCCGAGCTCTAATCAAGCTCGAAAACCAAACCCCTGTTTACAGGAACCAATACG

11427.2 GCCCACGTTGCTCTCGAGTTGAAGTGGGGCACC---------------------------

72791.2 ------------------------------------------------------------

236654.2 ------------------------------------------------------------

236612.2 ------------------------------------------------------------

105306.2 ------------------------------------------------------------

AB019195.1:1130-2451 GTCAAATGTTTGAGGCTTGCCCTGATGAGTTCCCCCAACTTCGGAGAACCGATGTGGCCA

reverse gccagatgtttgaagcgtgcccggatgaatttccgcagctgcagcgcaccaacgtggcgg

118981.2 ------------------------------------------------------------

100733.2 ------------------------------------------------------------

51475.2 ------------------------------------------------------------

140410.2 ------------------------------------------------------------

246005.2 GGCAGATGTTCGAGGCTTGCCCCGATGAATTCCCGCAACTCCAGAGAACCAATGTGGCCG

227217.2 GGCAGATGTTCGAGGCTTGCCCCGATGAATTCCCGCAACTCCAGAGAACCAATGTGGCCG

11427.2 -----ATCATTCCG-----CCTTGTTTGATGTCAACAATCGCAGCGGCCACATTGGTTCT

72791.2 ------------------------------------------------------------

236654.2 ------------------------------------------------------------

236612.2 ------------------------------------------------------------

105306.2 ------------------------------------------------------------

AB019195.1:1130-2451 CTTCCGTCGTCGATATCAAACAAGGTGGAATGATGGTGCCCCACTTCAACTCAAGAGCGA

reverse cggcgattgtggatattaaacagggcggcatgatggtgccgcattttaacagccgcgcga

118981.2 ------------------------------------------------------------

100733.2 ------------------------------------------------------------

51475.2 ------------------------------------------------------------

140410.2 ------------------------------------------------------------

246005.2 CTGCGATTGTTGACATCAAACAAGGCGGAATGATGGTG----------------------

227217.2 CTGCGATTGTTGACATCAAACAAGGCGGAATGATGGTGCCCCACTTCAACTCGAGAGCAA

11427.2 CTGGAGTTGCGGGAATTCATCGGGGC-------------AAGCCTCGAACATCTGC----

72791.2 -----------------------------------------------------------T

236654.2 ------------------------------------------------------------

236612.2 ------------------------------------------------------------

105306.2 ------------------------------------------------------------

AB019195.1:1130-2451 CATGGGTGGTGTTCGTTTCAGAAGGAGCTGGATCCTTCGAGATGGCCTGCCCTCACATAC

reverse cctgggcggtgtttgtggcggaaggcgcgggcagctttgaaatggcgtgcccgcatctgc

118981.2 ------------------------------------------------------------

100733.2 ------------------------------------------------------------

51475.2 ------------------------------------------------------------

140410.2 ------------------------------------------------------------

246005.2 ------------------------------------------------------------

227217.2 CGTGGGCGGTATTCGTTGCAGAAGGNGCCGGCTCCTTCGAGATGGCGTGCCCTNATCTTC

11427.2 ------CCGTATTGGTTGCTGTAAACGTGGG-----------------------------

72791.2 CTTAGGTTGTTTTGGGCGTTGATTC---CGA----------------------AGCCAAC

236654.2 ------------------------------------------------------------

236612.2 ------------------------------------------------------------

105306.2 ------------------------------------------------------------

AB019195.1:1130-2451 AGAGCAGCCAGTGGCAGCGAGGAAGGAGAGAGGAAGAACGACATTGGAGAAGGGAGGAAG

reverse gcggcgaagaatggcagcgcggccgccgcgaagaagaacgccattggcgccgcgaagaag

118981.2 ------------------------------------------------------------

100733.2 ------------------------------------------------------------

51475.2 ------------------------------------------------------------

140410.2 ------------------------------------------------------------

246005.2 ------------------------------------------------------------

227217.2 ------------------------------------------------------------

11427.2 ------------------------------------------------------------

72791.2 CAAGCGGAGATTCTCC-----TTCGGGGAAGCCATGACGGCGATTGGATGACCTGCCG--

236654.2 ------------------------------------------------------------

236612.2 ------------------------------------------------------------

105306.2 ------------------------------------------------------------

AB019195.1:1130-2451 AAGAGGAACGCGAAGAAAGAAGCGGTAGATTCGAAAGAGTTGCCGGTCGTCTATC-----

reverse aagaagaaagcgaaggccgcagcagccgctttgaacgcgtggcgggccatctgag-----

118981.2 ------------------------------------------------------------

100733.2 ------------------------------------------------------------

51475.2 ------------------------------------------------------------

140410.2 ------------------------------------------------------------

246005.2 ------------------------------------------------------------

227217.2 ------------------------------------------------------------

11427.2 ------------------------------------------------------------

72791.2 -----GGACGACGAGTA------AGCCGCCCGGTGATAGATGACCGGCGACTCTTTCAAA

236654.2 ------------------------------------------------------------

236612.2 ------------------------------------------------------------

105306.2 ------------------------------------------------------------

AB019195.1:1130-2451 -AGAGGGCGGCGTACTCGTAATTCCGGCAGGCCATCCAATCGCCATCATGGCTTC-CCCT

reverse -cccgggcggcctgctggtggtgccggcgggccatccgattgcggtgatggcgag-cccg

118981.2 -----------------------TCCGCGGCT----------------------------

100733.2 ------------------------------------------------------------

51475.2 ------------------------------------------------------------

140410.2 ------------------------------------------------------------

246005.2 ------------------------------------------------------------

227217.2 ------------------------------------------------------------

11427.2 ------------------------------------------------------------

72791.2 TCGACTGCTTCTTCCTTCTGATTCCTCG--------------------------------

236654.2 -ACCGGGCGGCTTACTCGTCGTCCCGGCAGGTCATCCAATCGCCGTCATGGCTTCC-CCG

236612.2 ---------------------------------ATCCAATCGCCGTCATGGC-TTCCCCG

105306.2 --------GGCTTACTCGTCGTCCCGGCAGGTCATCCAATCGCCGTCATGGCTTCCCCCG

AB019195.1:1130-2451 AATGAGAATCTCCGCTTGGTCGGGTTCGGAATCAATGCCGAAAACAACCACAGAAACTTC

reverse aaagaaaacctgcgcctggtgggctttggcattaacgcgcagaacaacctgcgcaacttt

118981.2 ------------------------------------------------------------

100733.2 ------------------------------------------------------------

51475.2 ------------------------------------GTCTAAAGCAGCTTCAAGAACATC

140410.2 ------------------------------------GTCTAAAGCAGCTTCAAGAACATC

246005.2 ------------------------------------------------------------

227217.2 ------------------------------------------------------------

11427.2 -----------------------GTTTGGTTTCGAG------------------------

72791.2 ------------------------------------------------------------

236654.2 AAGGAGAATCTCCGCTTGGTTGGCTTCGGAATCAACGCCCAAAACAACCTAAGAAACTTC

236612.2 AAGGAGAATCTCCGCTTGGTTGGCTTCGGAATCAACGCCCAAAACAACCTAAGAAACTTC

105306.2 AAGGAGAATCTCCGCTTGGTTGGCTTCGGAATCAACGCCCAAAACAACCTAAGAAACTTC

AB019195.1:1130-2451 CTCGCTGGGAGAGAGAACATAATGAACGAATTAGACAGAGAAGCAAAGGA----ACTTGC

reverse ctggcgggcaaagaaaacattatgaacgaagtggatcgcgaagcgaaaga----actgac

118981.2 ------------------------------------------------------------

100733.2 ------------------------------------------------------------

51475.2 ATTGCTGAAAACGG-TGTAATATGACTGATCTTCACCTCCAGCAGATAGGTAATCCTTAA

140410.2 ATTGCTGAAAACGG-TGTAATATGACTGATCTTCACCTCCAGCAGATAGGTAATCCTTAA

246005.2 ------------------------------------------------------------

227217.2 ------------------------------------------------------------

11427.2 ------------------------------------------------------------

72791.2 ------------------------------------------------------------

236654.2 CTAGCCGGGAAAGAGAACATAATGAACGAAGTAGACCAAGAAGCGAAGGA----ACTCAC

236612.2 CTAGCCGGGAAAGAGAACATAATGAACGAGGTAGACCGAGAAGCGAAGGA----ACTCAC

105306.2 CTAGCCGGGAAAGAGAACATAATGAACGAAGTAGACCAAGAAGCGAAGGA----ACTCAC

AB019195.1:1130-2451 CTTCAACGTAGAAGGAAAGCAAGCCGATGAGATATTCAGAAGCCAGAGAGAATCGTTCTT

reverse ctttaacattaaaggcaaagaagcggatgaaatttttaaaagccagcgcgaaagcttttt

118981.2 ------------------------------------------------------------

100733.2 ------------------------------------------------------------

51475.2 ACTCGCCGGGATTGTTAATGGGCTGAATCAA--TTTCACGATCTGTAGATCTTCATTCT-

140410.2 ACTCGCCGGGATTGTTAATGGGCTGAATCAA--TTTCACGATCTGTAGATCTTCATTCT-

246005.2 ------------------------------------------------------------

227217.2 ------------------------------------------------------------

11427.2 ------------------------------------------------------------

72791.2 ------------------------------------------------------------

236654.2 ATTCAACATAAAAGGAAAGGAAGCGGATGAGATTTTCAAAAGCCAGAGAGAATCGTTCTT

236612.2 ATTCAACATAAAAGGAAAGGAAGCGGATGAGATTTTCAAAAGCCAGAGAGAATCGTTCTT

105306.2 ATTCAACATAAAAGGAAAGGAAGCGGATGAGATTTTCAAAAGCCAGAGAGAATCGTTCTT

AB019195.1:1130-2451 CACAGAAGGGCCGGAAGGTGGTCGGAGGAGGTCGACGGAGAGAAGCCCGTTGTTGTCGAT

reverse taccaaaggcccggtgggcgaacgccgccgcagcaccgaacgccgcccgctgctgagcat

118981.2 ------------------------------------------------------------

100733.2 ------------------------------------------------------------

51475.2 CATGGTTGACCAAGTAAAT---CGTGGCCCCTGCCGGGATCACC----------------

140410.2 CATGGTTGACCAAGTAAAT---CGTGGCCCCTGCCGGGATCACCA---------------

246005.2 ------------------------------------------------------------

227217.2 ------------------------------------------------------------

11427.2 ------------------------------------------------------------

72791.2 ------------------------------------------------------------

236654.2 CACGAAAGGGCCGGTAGGGGAACGCCGGAGATCGACGGAGAGACGCCCGTTG--------

236612.2 CACGAAAGGGCCGGTAGGGGAACGCCGGAGATCGACGGAGAGACGCCCGTTGCTGTCGAT

105306.2 CACGAAAGGGCCGGTAGGGGAACGCCGGAGATCGACGGAGAGACGCCCGTTGCTGTCGAT

AB019195.1:1130-2451 TCTGAAACTGGCTGGTTACTTCTGAAA

reverse tctggaaagcgcgggctatctg-----

118981.2 ---------------------------

100733.2 ---------------------------

51475.2 ---------------------------

140410.2 ---------------------------

246005.2 ---------------------------

227217.2 ---------------------------

11427.2 ---------------------------

72791.2 ---------------------------

236654.2 ---------------------------

236612.2 TCTGGAATCGGCCGGTTACCTGTGAAA

105306.2 TC-------------------------

**Table S1**. Amino acid composition contig-deduced sequence of *Mc*7S.

|  | ***Momordica charantia*** | ***Solanum melongena*** | ***Capsicum annuum*** |
| --- | --- | --- | --- |
| **Amino Acid** | **No. of residues** | **No. of residues** | **No. of residues** |
| Ala (A) | 32 | 49 | 36 |
| Arg (R) | 38 | 22 | 20 |
| Asn (N) | 22 | 20 | 21 |
| Asp (D) | 14 | 14 | 11 |
| Cys (C) | 2 | 2 | 2 |
| Gln (Q) | 23 | 20 | 19 |
| Glu (E) | 42 | 24 | 29 |
| Gly (G) | 32 | 32 | 34 |
| His (H) | 12 | 10 | 5 |
| Ile (I) | 23 | 19 | 18 |
| Leu (L) | 31 | 24 | 25 |
| Lys (K) | 18 | 21 | 23 |
| Met (M) | 7 | 6 | 7 |
| Phe (F) | 21 | 29 | 27 |
| Pro (P) | 19 | 16 | 17 |
| Ser (S) | 27 | 23 | 19 |
| Thr (T) | 15 | 3 | 12 |
| Trp (W) | 2 | 1 | 1 |
| Tyr (Y) | 10 | 8 | 15 |
| Val (V) | 29 | 40 | 29 |

**Table S2.** The details of the H-bonds and salt bridges formed between N- and C-terminal domains of *Mc*7S.

| **H-bonds** | | | |
| --- | --- | --- | --- |
| **S.No.** | **N-terminal** | **Dist. [Å]** | **C-terminal** |
| 1 | N: VAL 143 [N] | 3.43 | C: ALA 249 [O] |
| 2 | N: VAL 141 [N] | 2.94 | C: ILE 251 [O] |
| 3 | N:ARG 138[NH2] | 3.14 | C:GLU 312[ OE2] |
| 4 | N:ASN  47[ND2] | 3.65 | C: ALA 340 [O] |
| 5 | N:TYR  50[OH] | 2.92 | C: SER 344 [O] |
| 6 | N:PHE  51[N] | 2.95 | C:LEU 348[O] |
| 7 | N:TYR  49[O] | 2.96 | C: VAL 350 [N] |
| 8 | N:TYR  49[OH] | 3.68 | C:HIS 355[ NE2] |
| 9 | N:TYR  50[OH] | 3.20 | C: SER 344 [N] |
| 10 | N:PHE  51[O] | 3.03 | C: LEU 348 [N] |
| 11 | N:GLU 107[OE1] | 2.37 | C:ARG 280[ NH1] |
| 12 | N:GLU 107[ OE2] | 3.82 | C:ARG 280[ NH2] |
| 13 | N:ASN 135[ OD1] | 3.84 | C:LYS 252[ NZ ] |
| 14 | N:GLY 139[O] | 3.06 | C:LEU 253[N] |
| 15 | N:GLY 139[O] | 3.12 | C:GLU 254[N] |
| 16 | N:ASP 140[OD1] | 3.06 | C:LYS 252[NZ] |
| 17 | N:ASP 140[OD2] | 3.04 | C:LYS 252[NZ] |
| 18 | N:VAL 141[O] | 2.75 | C:ILE 251[N] |
| **Salt bridge** | | | |
| **S.No.** | **N-terminal** | **Dist. [Å]** | **C-terminal** |
| 1 | N:ARG 138[NH2] | 3.14 | C:GLU 312[OE2] |
| 2 | N:GLU 107[OE1] | 3.77 | C:ARG 280[NH2] |
| 3 | N:GLU 107[OE1] | 2.37 | C:ARG 280[NH1] |
| 4 | N:GLU 107[OE2] | 3.82 | C:ARG 280[NH2] |
| 5 | N:GLU 107[OE2] | 3.72 | C:ARG 280[NH1] |
| 6 | N:GLU 137[OE2] | 2.91 | C:LYS 252[NZ] |
| 7 | N:ASP 140[OD1] | 3.06 | C: LYS 252 [NZ] |
| 8 | N:ASP 140[OD2] | 3.04 | C:LYS 252[NZ] |

**Table S3.** The details of the H-bonds and salt bridges formed between chain A-chain B and chain C-chain A of *Mc*7S.

| **Hydrogen bond chain A-chain B** | | | |
| --- | --- | --- | --- |
| **S. No.** | **Chain B** | **Dist. [Å]** | **Chain A** |
| 1 | B:HIS 103[NE2] | 3.19 | A:GLY 354[O] |
| 2 | B:GLN 124[N] | 2.52 | A:ASN 382[OD1] |
| 3 | B:GLN 124[NE2] | 2.97 | A:GLU 388[O] |
| 4 | B:ARG 131[NH2] | 2.25 | A:ASP 395[OD2] |
| 5 | B:TYR 151[OH] | 2.27 | A:ASP 395[OD2] |
| 6 | B:ASN 170[ND2] | 3.37 | A:ALA 353[O] |
| 7 | B:ASN 171[ND2] | 2.87 | A:ASP  78[O] |
| 8 | B:LYS 176[NZ] | 3.69 | A:PRO 322[O] |
| 9 | B:TYR 189[OH] | 3.01 | A:LEU 384[O] |
| 10 | B:SER 193[N] | 3.27 | A:GLU 318[OE1] |
| 11 | B:SER 193[OG] | 2.44 | A:GLU 318[OE2] |
| 12 | B:ARG 207[NH2] | 3.74 | A:GLU 412[OE1] |
| 13 | B:GLU 107[OE2] | 3.46 | A:ARG 303[NH1] |
| 14 | B:GLN 124[OE1] | 2.79 | A:PHE 383[N] |
| 15 | B:ALA 146[O] | 3.20 | A:SER 302[OG] |
| 16 | B:GLY 147[O] | 3.37 | A:ASN 382[ND2] |
| 17 | B:TYR 151[OH] | 3.39 | A:ASP 395[N] |
| 18 | B:ASN 170[OD1] | 3.41 | A:THR 305[OG1] |
| 19 | B:ASN 170[OD1] | 3.27 | A:ALA 353[N] |
| 20 | B:ALA 199[O] | 3.22 | A:GLN 417[NE2] |
| 21 | B:ALA 200[O] | 2.92 | A:GLN 417[NE2] |
| 22 | B:LEU 201[O] | 3.65 | A:GLN 417[N] |
| 23 | B:ASP 202[O] | 2.90 | A:SER 416[OG] |
| 24 | B:ARG 210[O] | 3.46 | A:ASN 404[ND2] |
| 25 | B:VAL 211[O] | 3.42 | A:ASN 404[N] |
| **Salt bridge chain A-chain B** | | | |
| **S. No.** | **Chain B** | **Dist. [Å]** | **Chain A** |
| 1 | B:ARG 131[NE] | 3.65 | A:ASP 395[OD2] |
| 2 | B:ARG 131[NH2] | 2.25 | A:ASP 395[OD2] |
| 3 | B:ARG 207[NH2] | 3.74 | A:GLU 412[OE1] |
| 4 | B:GLU 107[OE2] | 3.46 | A:ARG 303[NH1] |
| **Hydrogen bond chain C-chain A** | | | |
| S. No. | Chain C | Dist. [Å] | [Chain A](javascript:openWindow('pi_ipage_atom2.html',400,250);) |
| 1 | C:ARG 303[NH1] | 3.46 | A:GLU 107[OE2] |
| 2 | C:PHE 383[N] | 2.79 | A:GLN 124[OE1] |
| 3 | C:SER 302[OG] | 3.20 | A:ALA 146[O] |
| 4 | C:ASN 382[ND2] | 3.37 | A:GLY 147[O] |
| 5 | C:ASP 395[N] | 3.39 | A:TYR 151[OH] |
| 6 | C:THR 305[OG1] | 3.41 | A:ASN 170[OD1] |
| 7 | C:ALA 353[N] | 3.27 | A:ASN 170[OD1] |
| 8 | C:GLN 417[NE2] | 3.22 | A:ALA 199[O] |
| 9 | C:GLN 417[NE2] | 2.92 | A:ALA 200[O] |
| 10 | C:GLN 417[N] | 3.65 | A:LEU 201[O] |
| 11 | C:SER 416[OG] | 2.90 | A:ASP 202[O] |
| 12 | C:ASN 404[ND2] | 3.46 | A:ARG 210[O] |
| 13 | C:ASN 404[ N  ] | 3.42 | A:VAL 211[ O  ] |
| 14 | C:GLY 354[ O  ] | 3.19 | A:HIS 103[ NE2] |
| 15 | C:ASN 382[ OD1] | 2.52 | A:GLN 124[ N  ] |
| 16 | C:GLU 388[ O  ] | 2.97 | A:GLN 124[ NE2] |
| 17 | C:ASP 395[ OD2] | 2.25 | A:ARG 131[ NH2] |
| 18 | C:ASP 395[ OD2] | 2.27 | A:TYR 151[ OH ] |
| 19 | C:ALA 353[ O  ] | 3.37 | A:ASN 170[ ND2] |
| 20 | C:ASP  78[ O  ] | 2.87 | A:ASN 171[ ND2] |
| 21 | C:PRO 322[ O  ] | 3.69 | A:LYS 176[ NZ ] |
| 22 | C:LEU 384[ O  ] | 3.01 | A:TYR 189[ OH ] |
| 23 | C:GLU 318[ OE1] | 3.27 | A:SER 193[ N  ] |
| 24 | C:GLU 318[ OE2] | 2.44 | A:SER 193[ OG ] |
| 25 | C:GLU 412[ OE1] | 3.74 | A:ARG 207[ NH2] |
| **Salt bridge chain C-chain A** | | | |
| S. No. | Chain C | Dist. [Å] | [Chain A](javascript:openWindow('pi_ipage_atom2.html',400,250);) |
| 1 | C:ARG 303[NH1] | 3.46 | A:GLU 107[OE2] |
| 2 | C:ASP 395[OD2] | 3.65 | A:ARG 131[NE] |
| 3 | C:ASP 395[OD2] | 2.25 | A:ARG 131[NH2] |
| 4 | C:GLU 412[OE1] | 3.74 | A:ARG 207[NH2] |

**Table S4.** The net intensity mean and mean concentration values of analyte (Cu^2+^) in the sample as obtained from ICP-MS analysis of the *Mc*7S sample and control sample.

| **Analyte/Mass** |  | **Net Intens. Mean** | **Conc. Mean (in ppb)** |
| --- | --- | --- | --- |
| Cu  63 | Sample | 119968.318 | 25.161 |
|  | 10 ppm control | 40564.178 | 10.000 |
|  | 100 ppm control | 358980.315 | 99.871 |
|  | 1000 ppm control | 4779905.019 | 1002.486 |

**Table S5.** Molecular docking analysis of ACE with LPR and PEP (VFK-peptide) using HADDOCK.

| **S. No.** | **Analysis Component** | **ACE-LPR** | **ACE-PEP** |
| --- | --- | --- | --- |
| 1 | HADDOCK score | -73.5 +/-0.5 | -81.2 +/- 3.8 |
| 2 | Cluster size | 115 | 152 |
| 3 | RMSD from the overall lowest-energy structure | 0.3 +/-0.2 | 0.2 +/- 0.1 |
| 4 | Van der Waals energy (kcal mol^-1^) | -25.0 +/-6.0 | -28.8 +/- 2.7 |
| 5 | Electrostatic energy (kcal mol^-1^) | -247.7 +/- 38.8 | -205.6 +/ 13.2 |
| 6 | Desolvation energy (kcal mol^-1^) | -0.7 +/-0.2 | -16.8 +/- 4.4 |
| 7 | Restraints violation energy (kcal mol^-1^) | 16.5 +/- 2.03 | 54.7 +/- 15.52 |
| 8 | Buried surface area (Å^2^) | 843.3 +/- 60.9 | 917.4 +/- 15.5 |
| 9 | Z-score | -1.4 | -1.7 |

**Table S6.** Chemical reactive properties (HOMO, LUMO, energy gap, electro-negativity, hardness and softness) predicted by DFT calculations of LPR and PEP.

| **S. No.** | **Compound** | **HOMO (eV)** | **LUMO (eV)** | **Energy Gap (eV)** | **Electro**  **Negativity (eV)** | **Hardness (eV)** | **Softness (eV)** |
| --- | --- | --- | --- | --- | --- | --- | --- |
| 1 | LPR | -6.56 | -5.03 | 1.53 | 5.79 | 0.765 | 1.31 |
| 2 | PEP | -6.45 | -1.97 | 4.48 | 4.21 | 2.24 | 0.44 |

**Table S7.** The average RMSD, radius of gyration, SASA and intra-H bond numbers for ACE-LPR and ACE-PEP complexes at 300K.

| **S.No.** | **Compounds** | **Average Protein RMSD (nm)** | **Average Radius of gyration (nm)** | **Average SASA**  **(nm)^2^** | **Intra-H bond numbers** |
| --- | --- | --- | --- | --- | --- |
| 1. | ACE-LPR | 0.25 | 2.40744 | 259.90 | 479.63 |
| 2. | ACE-PEP | 0.25 | 2.403473 | 261.14 | 480.86 |


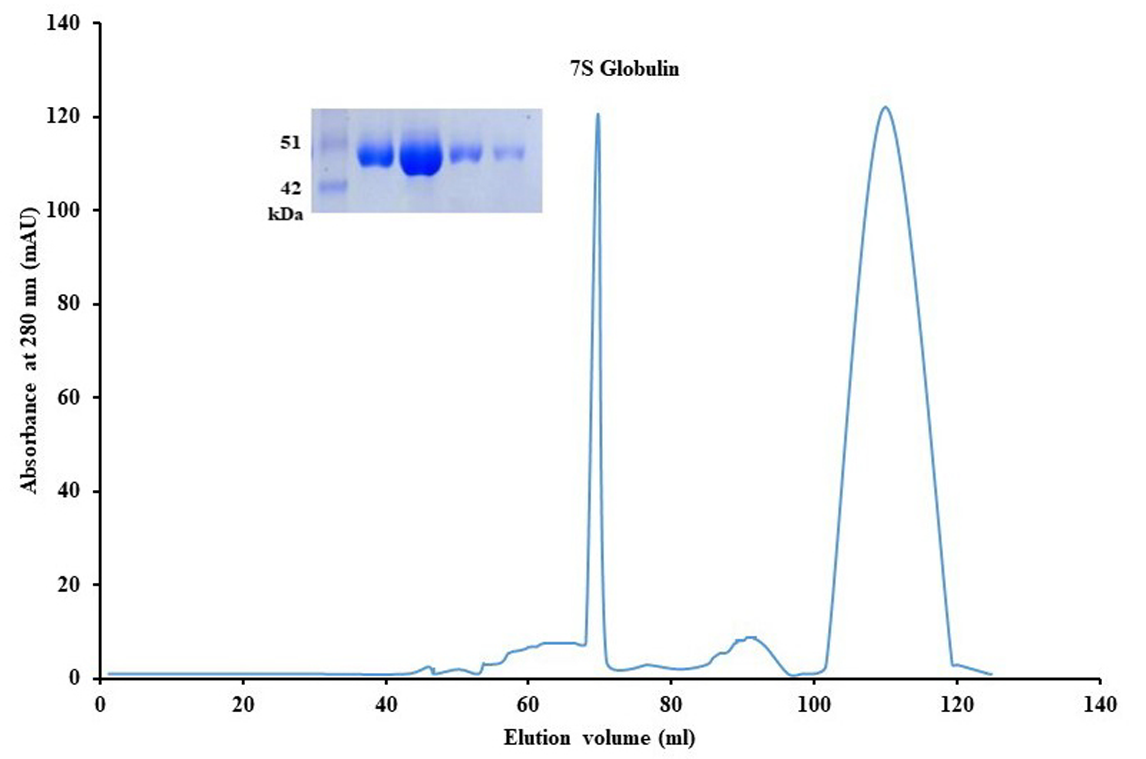


**Fig. S1.** Gel filtration profile of *Momordica charantia*7S globulin. Chromatogram of sample after size exclusion chromatography (HiLoad 16/60 Superdex 200 pg column) step; peak fraction at elution volume ~69.2 ml correspond to the purified fraction of *Mc*7S globulin.


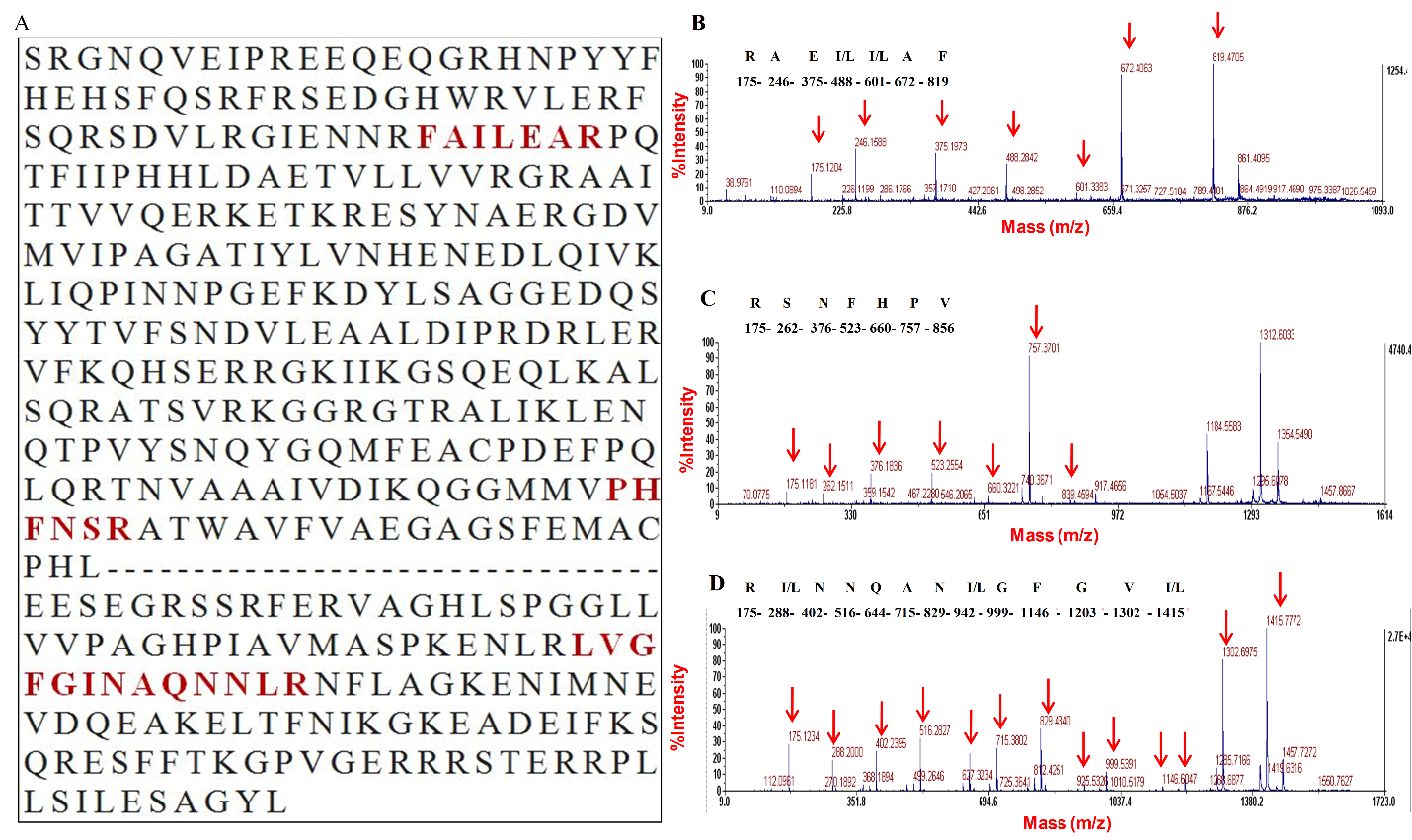


**Fig. S2.** The sequence of *Momordica charantia* 7S globulin and peptide fragment derived from MALDI-ESI-MS results. (A) The protein sequence of *Mc*7S globulin derived by aligning of contig fragments deposited in the GenBank Short Read Archive (SRA) with the accession number SRP004091. The accession numbers for the individual experiments of normalized sequence data is SRX030203. Regions underlined represent the three internal peptide sequences obtained from partial internal sequencing results. Peptide fragment from the ESI-MS. Three peptide fragments FAILEAR (B), PHFNSR (C) and LVGFGINAQNNLR (D) were identified from MALDI-ESI-MS after digestion of *Mc*7S globulin protein.


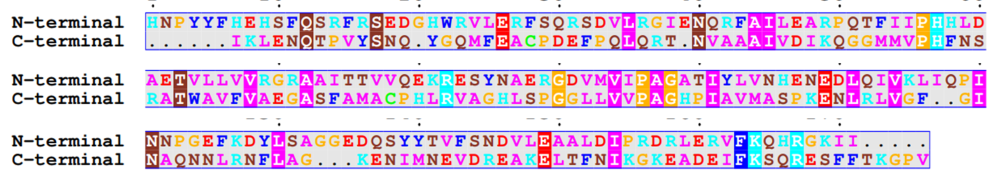


**Fig. S3.** The sequence alignment of N- and C-terminal domain of *Mc*7S share high sequence homology. The residues have been coloured as per properties.


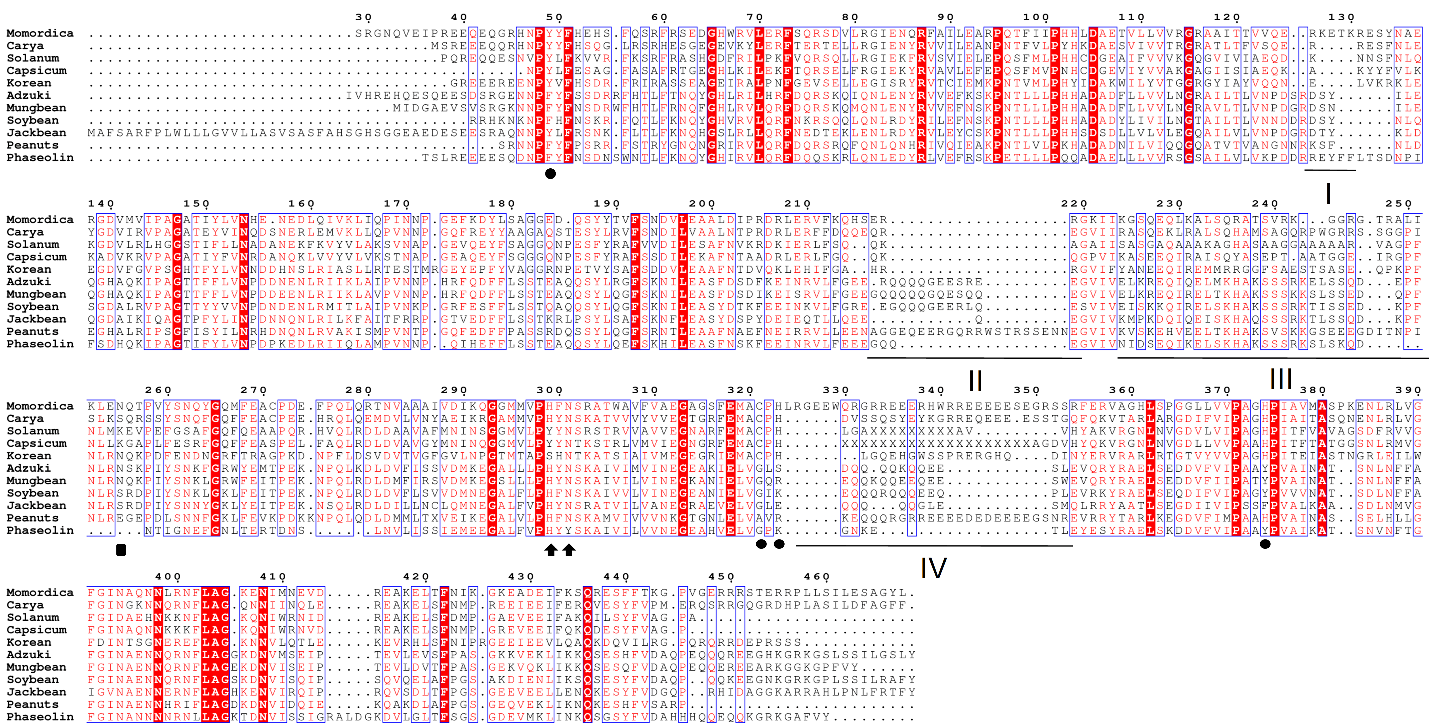


**Fig. S4.** Multiple sequence alignment of 7S globulins or vicilins deposited in the PDB. The pBLAST search against 7S globulin sequence suggests that *Mc*7S structure share highest similarity with vicilin from pecan, *Capsicum annum* Vic_CAPAN and *Solanum melongena* SM80.1, followed by Korean pine, 7S globulin-3 adzuki bean, 8S mungbean, soybean, peanut, french bean and jackbean. Copper binding and acetate binding residues of *Mc*7S are shown by black circles and arrow, respectively. The probable N-glycosylation site is shown by black box. The length of disorder loop I, II, III and IV regions has been marked by line.

**
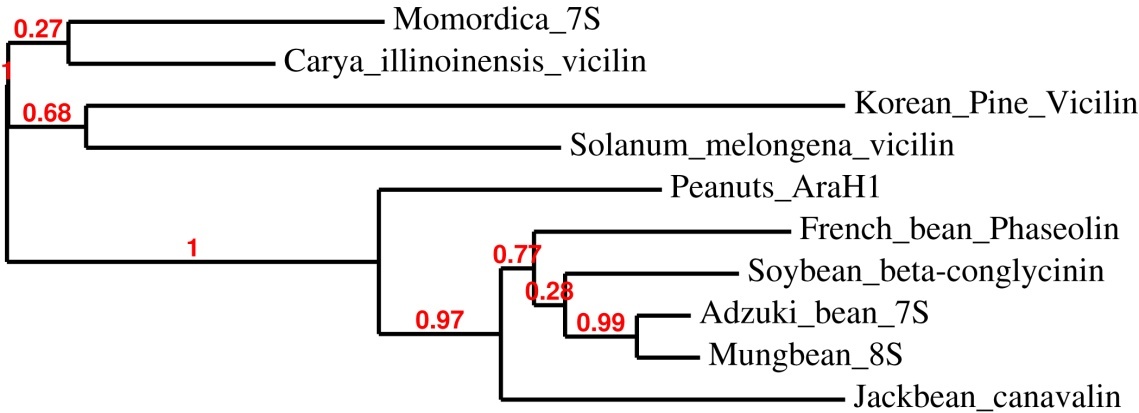
**

**Fig. S5.** Maximum likelihood (ML) phylogenetic tree constructed using sequences of *Mc*7S, and other known vicilins like pecan allergen (*Carya illinoinensis*), *Solanum melongena* SM80.1, Korean pine vicilin, β-conglycinin from soybean, phaseolin from French bean, 7S globulin-1 adzuki bean, 8S mungbean, canavalin from Jackbean and vicilin AraH1 from *Arachis hypogaea*.


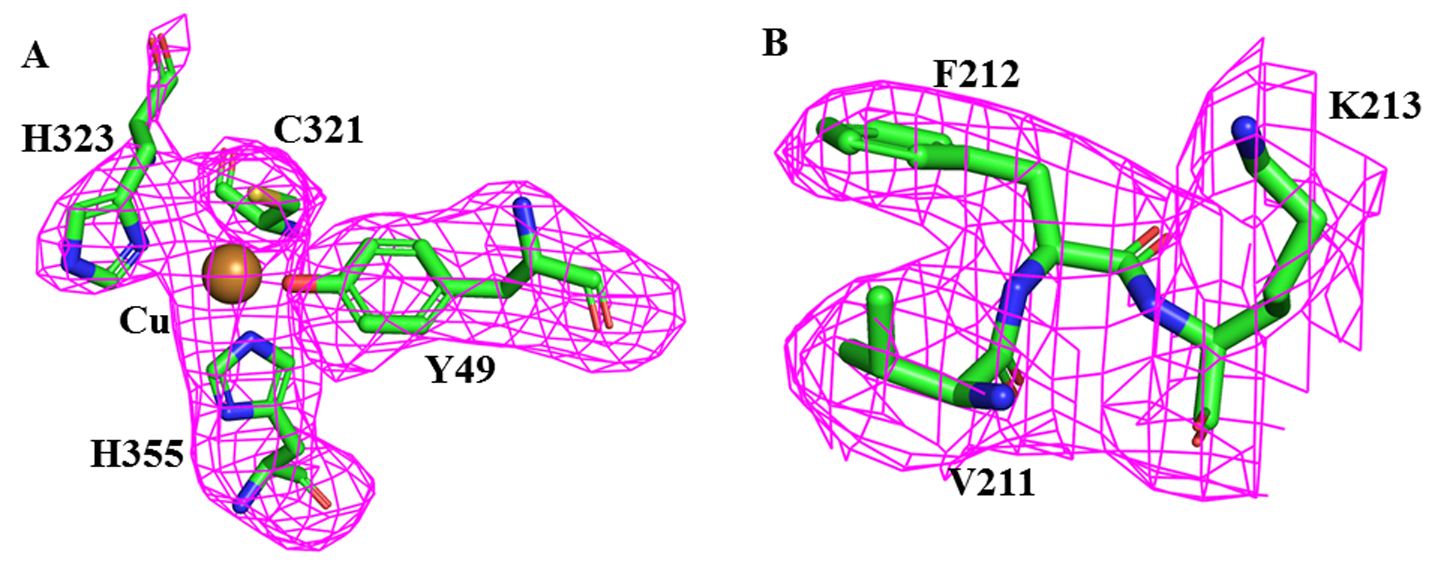


**Fig. S6.** Polder omit maps (mFo-DFc) at 2.5 σ for Copper (Cu) and VFK tripeptide of *Mc*7S. A) Cu and its coordinating residues are shown in sphere and stick format, respectively. B) Omit map of V^211^F^212^K^213^ tripeptide in which residues are shown in stick format in green color while omit map is represented in magenta color.


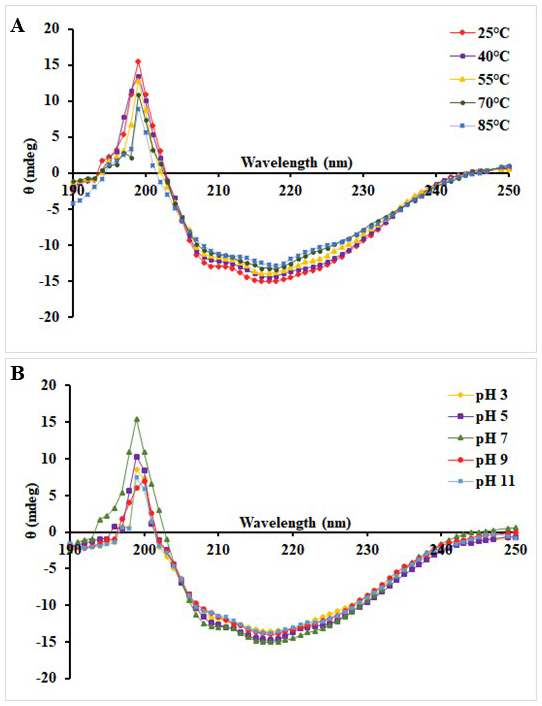


**Fig. S7.** Effect of temperature and pH variation on secondary structure of *Momordica charantia* 7S globulin. (A) Far-UV CD spectrum from (190 to 250 nm) of *Mc*7S globulin at different temperatures (25-85°C). (B) Far-UV CD spectrum from (190 to 250 nm) of *Mc*7S globulin at different pH (3-11).The CD spectrum was recorded at protein concentration of 0.2 mg/ml in 1 mm quartz cell. CD spectrum showed Spectra showed a small negative peak near 208 nm, positive peak between 195 nm and 200 nm and broad negative peak between 212-220 nm which are the characteristic features of protein having predominant β-sheet with little α-helix.

**
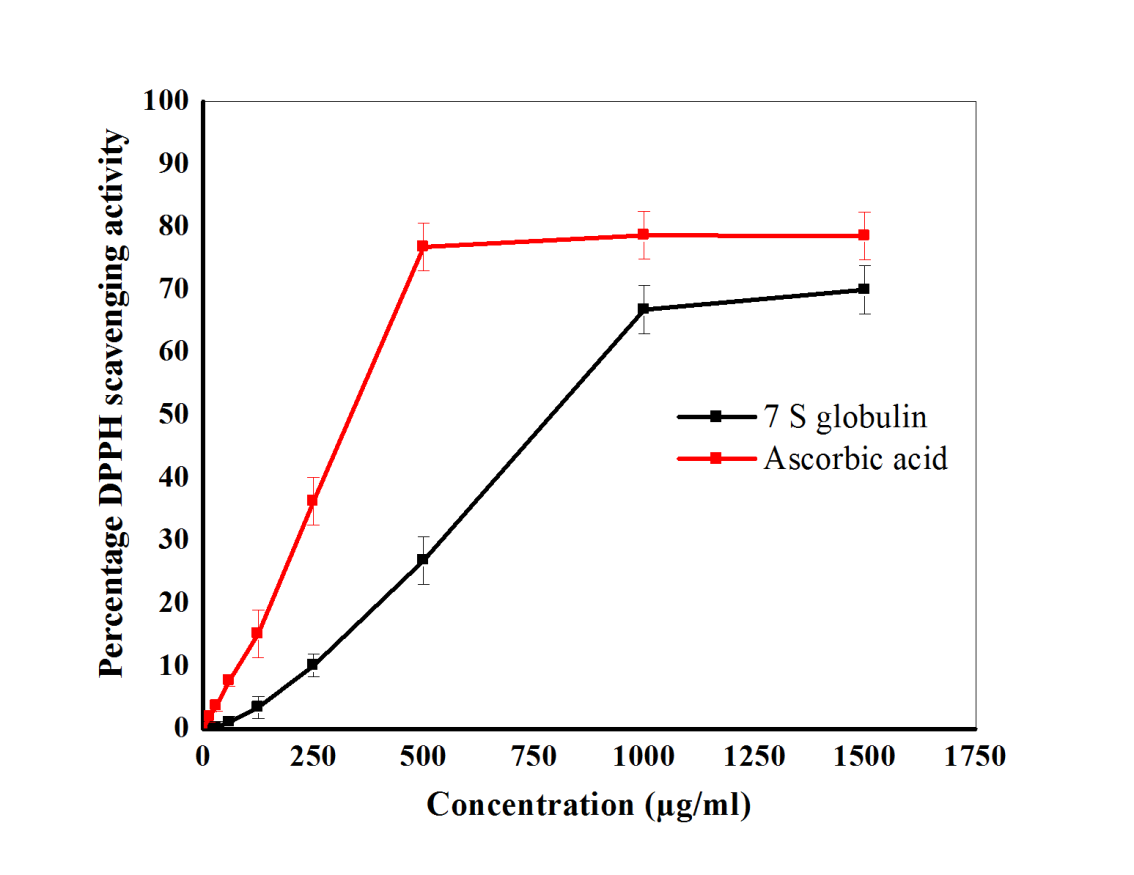
**

**Fig. S8.** Effect of *Mc*7s globulin (red) and Ascorbic acid (black) on DPPH (2,2-Diphenyl-1-picrylhydrazyl) radical scavenging activity.


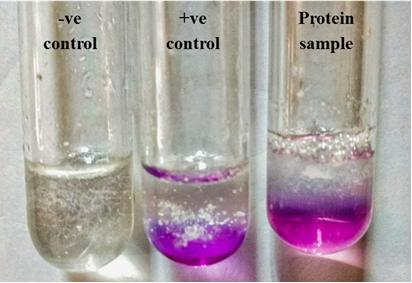


**Fig. S9**. Molisch’s test:- positive control having only buffer, positive control having glucose with buffer, protein sample having *Mc*7S globulin with buffer.

**References**

1 Natesh, R., Schwager, S. L., Sturrock, E. D. & Acharya, K. R. Crystal structure of the human angiotensin-converting enzyme–lisinopril complex. *Nature* 421, 551 (2003).

2 Kurkcuoglu, Z. *et al.* Performance of HADDOCK and a simple contact-based protein–ligand binding affinity predictor in the D3R Grand Challenge 2. *Journal of computer-aided molecular design* 32, 175-185 (2018).

3 Van Zundert, G. *et al.* The HADDOCK2. 2 web server: user-friendly integrative modeling of biomolecular complexes. *Journal of molecular biology* 428, 720-725 (2016).

4 DeLano, W. The PyMOL Molecular Graphics System on World Wide Web <http://www>. pymol. org. (2002).

5 Laskowski, R. A. & Swindells, M. B. (ACS Publications, 2011).

6 Becke, A. D. Density‐functional thermochemistry. III. The role of exact exchange. *The Journal of chemical physics* 98, 5648-5652 (1993).

7 Frisch, M. *et al.* Gaussian 16, Revision A. 03, Gaussian. *Inc., Wallingford CT* (2016).

8 Lee, C., Yang, W. & Parr, R. G. Development of the Colle-Salvetti correlation-energy formula into a functional of the electron density. *Physical review B* 37, 785 (1988).

9 Schlegel, H. B. Optimization of equilibrium geometries and transition structures. *Journal of Computational Chemistry* 3, 214-218 (1982).

10 Van Der Spoel, D. *et al.* GROMACS: fast, flexible, and free. *Journal of computational chemistry* 26, 1701-1718 (2005).

11 van Gunsteren, W. F. *et al.* Biomolecular simulation: the {GROMOS96} manual and user guide. (1996).

12 Schüttelkopf, A. W. & Van Aalten, D. M. PRODRG: a tool for high-throughput crystallography of protein–ligand complexes. *Acta Crystallographica Section D: Biological Crystallography* 60, 1355-1363 (2004).

13 Abraham, M. J. & Gready, J. E. Optimization of parameters for molecular dynamics simulation using smooth particle‐mesh Ewald in GROMACS 4.5. *Journal of computational chemistry* 32, 2031-2040 (2011).
